# Platelet-rich plasma in cartilage repair: biological mechanisms and clinical perspectives

**DOI:** 10.3389/fspor.2026.1785754

**Published:** 2026-04-29

**Authors:** Mingzhu Li, Chao Jing, Qiaofeng Liu, Yu Zhang, Kainan Li

**Affiliations:** 1 Department of Orthopedics, Sichuan Taikang Hospital, Chengdu, China; 2 Department of Sports Rehabilitation and Pain Medicine, Sports Science Research Institute of Hebei Province, Shijiazhuang, China; 3 School of Pharmaceutical Sciences, Fudan University, Shanghai, China; 4 College of Nursing, Youjiang Medical University for Nationalities, Baise, China

**Keywords:** cartilage, osteoarthritis, platelet-rich plasma, signaling pathways, therapeutic

## Abstract

Recent studies indicate that platelet-rich plasma (PRP) is receiving growing attention as a biologically based intervention for cartilage damage and osteoarthritis. Its proposed therapeutic potential is attributed to its capacity to modulate multiple molecular pathways involved in cartilage metabolism and inflammatory responses. Evidence from *in vitro* and animal studies suggests that PRP may affect signaling pathways, including PI3K/AKT/mTOR, NF-κB, Wnt/β-catenin, and TGF-β/Smad, all of which are implicated in autophagy regulation, inflammatory modulation, and extracellular matrix homeostasis. Nevertheless, most of the mechanistic evidence currently comes from preclinical models, whereas high-quality clinical data validating these biological effects in patients remain scarce. Consequently, the translational relevance of these mechanisms to clinical outcomes requires further clarification. Standardized PRP preparation methods, well-defined dosing strategies, and robust long-term studies evaluating clinical safety and efficacy are essential before PRP can be reliably incorporated into routine regenerative orthopedic practice.

## 1 Introduction

Articular hyaline cartilage is a highly specialized connective tissue that provides a smooth, lubricated surface for articulation and facilitates load distribution within synovial joints (1). Because articular cartilage is avascular, aneural, and alymphatic, it depends primarily on diffusion from the synovial fluid and secondarily from the subchondral bone for its supply of oxygen and nutrients. In human articular cartilage, chondrocytes occupy approximately only 1.65% of the total tissue volume, with the remaining volume consisting of the extracellular matrix (ECM). The ECM is a highly hydrated network composed predominantly of collagens, especially type II collagen, and proteoglycans (PRG), which together provide hyaline cartilage with its characteristic mechanical and biochemical properties (2).

Articular cartilage injury may arise from acute trauma, chronic joint instability leading to abnormal mechanical loading, repetitive overuse in athletic activity, obesity-associated overload, or age-related degenerative processes (3). Osteoarthritis (OA), one of the most common degenerative joint diseases and affecting more than 240 million people worldwide, represents a major clinical consequence of articular cartilage damage. Its increasing prevalence is a substantial contributor to pain, functional impairment, and disability on a global scale. In OA, cartilage degeneration develops through a series of interrelated pathological processes, typically including early ECM breakdown, localized inflammation, and upregulation of matrix-degrading enzymes such as matrix metalloproteinases (MMPs) (4).

**Figure 1:**
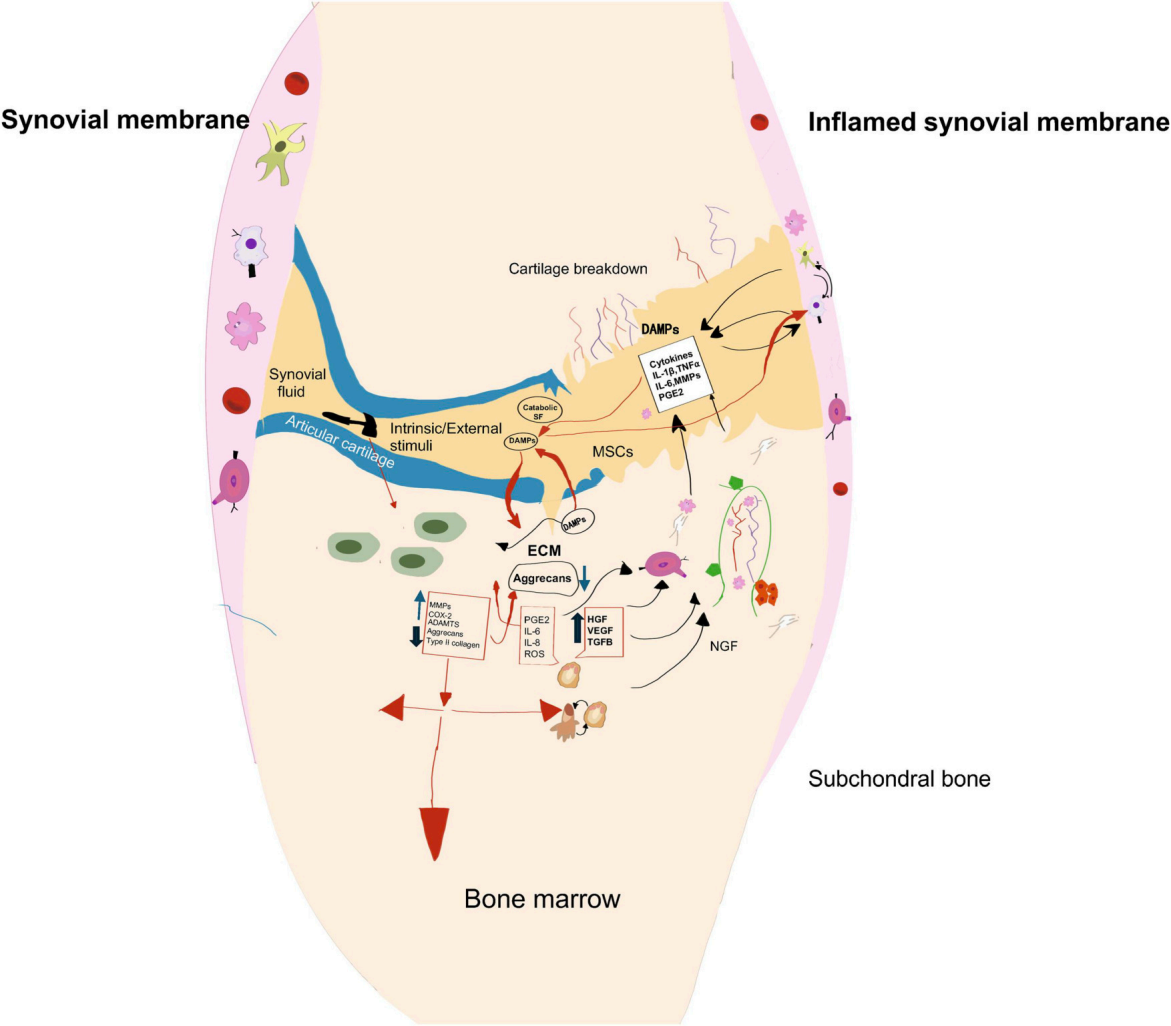
Mechanisms of mechanical loading–induced cartilage degeneration and synovitis. Abnormal mechanical load distribution on articular cartilage disrupts its homeostatic equilibrium, triggering adaptive or catabolic responses in chondrocytes. This process leads to an increased synthesis of matrix metalloproteinases (MMPs) and adenosine diphosphate nicotinamide adenine dinucleotide synthase (ADAMTS), promoting the expression of proinflammatory cytokines and mediators such as interleukin-1β (IL-1β) and cyclooxygenase-2 (COX-2), and other proinflammatory cytokines and mediators, accompanied by elevated reactive oxygen species production, disrupted tissue hydration, and matrix fragmentation. Proinflammatory cytokines such as IL-1β and TNF-α play a central role in cartilage destruction: they not only inhibit glycosaminoglycan and type II collagen synthesis but also directly stimulate chondrocyte MMP production. Furthermore, damage-associated molecular patterns originating from the inflammatory environment activate toll-like receptors (TLRs) on the surfaces of synovial macrophages, fibroblasts, and monocytes, thereby exacerbating synovitis through the NF-κB signaling pathway. These activated cells release a cascade of catabolic mediators, including MMP-1, MMP-3, MMP-13, IL-1β, TNF-α, and IL-6, collectively driving the onset and progression of synovitis in osteoarthritis.

Mechanical loading is a critical regulator of joint homeostasis; however, excessive, repetitive, or abnormal loading can drive cartilage and synovium from a physiological state toward a pathological one. In parallel, abnormal loading promotes synovial activation and the release of inflammatory mediators, which further exacerbate cartilage catabolism. Consequently, mechanical overloading contributes to osteoarthritis progression through a whole-joint pathogenic process characterized by reciprocal amplification among cartilage degeneration, synovitis, and biomechanical imbalance (Figure 1). In this setting, platelet-rich plasma (PRP), an autologous blood-derived product enriched in platelets and growth factors, has gained considerable attention owing to its biological activity, favorable safety profile as an autologous preparation, and the relative simplicity of its preparation (5). Preclinical and clinical evidence suggests that PRP may exert therapeutic effects on cartilage pathology by modulating inflammatory responses, supporting extracellular matrix synthesis, and improving pain and functional outcomes in patients with joint disorders (6, 7). Experimental studies have also indicated that PRP may promote osteochondral graft integration at the articular surface and attenuate degenerative changes, as demonstrated by higher histological scores and less severe cartilage damage in treated joints (8). PRP appears to exert its effects on articular cartilage, synovial tissue, and subchondral bone through the modulation of multiple signaling pathways, including phosphoinositide 3-kinase/protein kinase B/mammalian target of rapamycin (PI3K/AKT/mTOR), Wnt/β-catenin, nuclear factor-κB (NF-κB), and transforming growth factor-β (TGF-β)/Smad (9, 10). PRP is also increasingly being explored as a biological adjunct to surgical procedures for cartilage and ligament repair.

This narrative review aims to provide a structured overview of the current understanding of PRP therapy for articular hyaline cartilage damage, with a particular focus on its clinical applications in joint disorders such as OA. We first summarize the principal mechanistic insights into how PRP may modulate relevant signaling pathways in cartilage and periarticular tissues and then examine recent advances in clinical application, including PRP preparation protocols, dosing strategies, and reported outcomes. Finally, we identify existing gaps in the evidence, outline priorities for future research, and discuss important considerations for the design of clinical trials required to more clearly establish the role of PRP in the management of articular cartilage lesions.

## 2 Materials and methods

To provide a comprehensive yet critical overview, we searched PubMed, Web of Science, and Embase databases from January 2000 to December 2025. Search terms included combinations of “platelet-rich plasma OR PRP,” “cartilage OR cartilage injury AND platelet-rich plasma OR PRP,” “osteoarthritis AND platelet-rich plasma,” “signaling pathway AND platelet-rich plasma,” “PI3K/AKT/mTOR AND platelet-rich plasma,” “NF-kappa B AND platelet-rich plasma,” “Wnt/β-catenin AND platelet-rich plasma,” and “TGF-β/Smad AND platelet-rich plasma.” We included original research articles (*in vitro*, *in vivo*, and clinical studies) that investigated PRP effects on cartilage or chondrocytes in the context of the above pathways. Reviews, case reports, and non-English articles were excluded. This review aims to synthesize how PRP specifically modulates these pathways, critically appraise the evidence, and identify knowledge gaps to guide future research and clinical practice.

## 3 Pathogenic pathways and molecules

The preservation of joint cartilage homeostasis is a complex biological process governed by an intricate network of signaling pathways. Mechanical or biological insults can disrupt the ECM and impair chondrocyte function, thereby reducing the synthesis of type II collagen and proteoglycans. Concomitantly, local inflammation develops, characterized by elevated levels of proinflammatory cytokines such as interleukin-1β (IL-1β) and tumor necrosis factor-α (TNF-α), together with increased expression of MMPs. These mediators accelerate ECM degradation and disturb the balance between matrix synthesis and breakdown, leading to progressive cartilage thinning, fibrillation, and, if the process remains uncontrolled, full-thickness cartilage defects. In advanced stages, exposure of the subchondral bone contributes to joint pain, stiffness, and functional impairment, ultimately manifesting clinically as OA.

Several keys signaling pathways—including Wnt/β-catenin, NF-κB, PI3K/AKT/mTOR, and TGF-β/Smad—have been widely investigated for their roles in cartilage development, homeostasis, degeneration, and repair. A more comprehensive understanding of how dysregulation of these pathways drives OA pathogenesis not only refines the current knowledge of disease mechanisms but also identifies potential molecular targets for therapeutic intervention aimed at cartilage preservation and regeneration. In the following sections, we summarize current insights into these major signaling pathways and discuss their relevance to OA pathogenesis and treatment, with particular emphasis on how PRP may influence these pathways in the setting of articular cartilage damage.

### 3.1 Wnt signaling pathway

The Wnt signaling pathway is a fundamental regulator of embryonic development, controlling cell proliferation, differentiation, polarity, and fate determination. In skeletal development, it plays a key role in the differentiation of osteoblasts and chondrocytes. In postnatal articular cartilage, Wnt signaling is generally maintained in a tightly regulated low-activity state, which is essential for preserving the quiescent chondrocyte phenotype and sustaining cartilage homeostasis.

The Wnt signaling network is broadly classified into two major branches based on its dependence on β-catenin: the canonical (β-catenin-dependent) pathway and the non-canonical (β-catenin-independent) pathway (11). In the canonical pathway, the binding of Wnt proteins (e.g., Wnt1 and Wnt3a) to the cell-surface receptors Frizzled and LRP5/6 suppresses the activity of the destruction complex, which is composed of proteins such as Axin and glycogen synthase kinase (GSK)-3β. Consequently, β-catenin escapes degradation, accumulates in the cytoplasm, and translocates into the nucleus. In the nucleus, β-catenin associates with T-cell factor/lymphoid enhancer factor (TCF/LEF) transcription factors to activate specific target genes, including c-Myc and Cyclin D1, thereby regulating the proliferation, differentiation, and survival of chondrocytes (12).

Genome-wide association studies have identified genetic loci associated with OA risk, including variants in the *FRZB* gene, which encodes secreted frizzled-related protein 3, a Wnt inhibitor, and in the *DOT1L* gene, which regulates the expression of Wnt target genes (15, 16). β-Catenin expression is markedly upregulated in cartilage from patients with OA. Experimental models have highlighted the critical importance of tightly controlled Wnt/β-catenin signaling in the maintenance of articular cartilage. Tong et al. demonstrated that overexpression of ICAT (inhibitor of β-catenin and TCF) in Col2a1-ICAT transgenic mice induced articular cartilage degeneration associated with increased chondrocyte apoptosis, suggesting that excessive suppression of Wnt/β-catenin signaling is harmful to cartilage homeostasis (13). Conversely, genetic deletion of Wnt9a in mice led to joint abnormalities and synovial chondroid metaplasia, which were further aggravated by concomitant loss of Wnt4, suggesting that multiple Wnt ligands fulfill non-redundant and essential functions in joint development and maintenance (14). Furthermore, pharmacological activation of β-catenin signaling through intra-articular injection of lithium chloride (LiCl) in rabbits induced cartilage degeneration and was associated with increased expression of MMP-13 and p53, thereby promoting OA progression (17). Taken together, these findings suggest that both inadequate and excessive activation of canonical Wnt signaling can disturb cartilage homeostasis and thereby contribute to OA pathogenesis.

**Figure 2:**
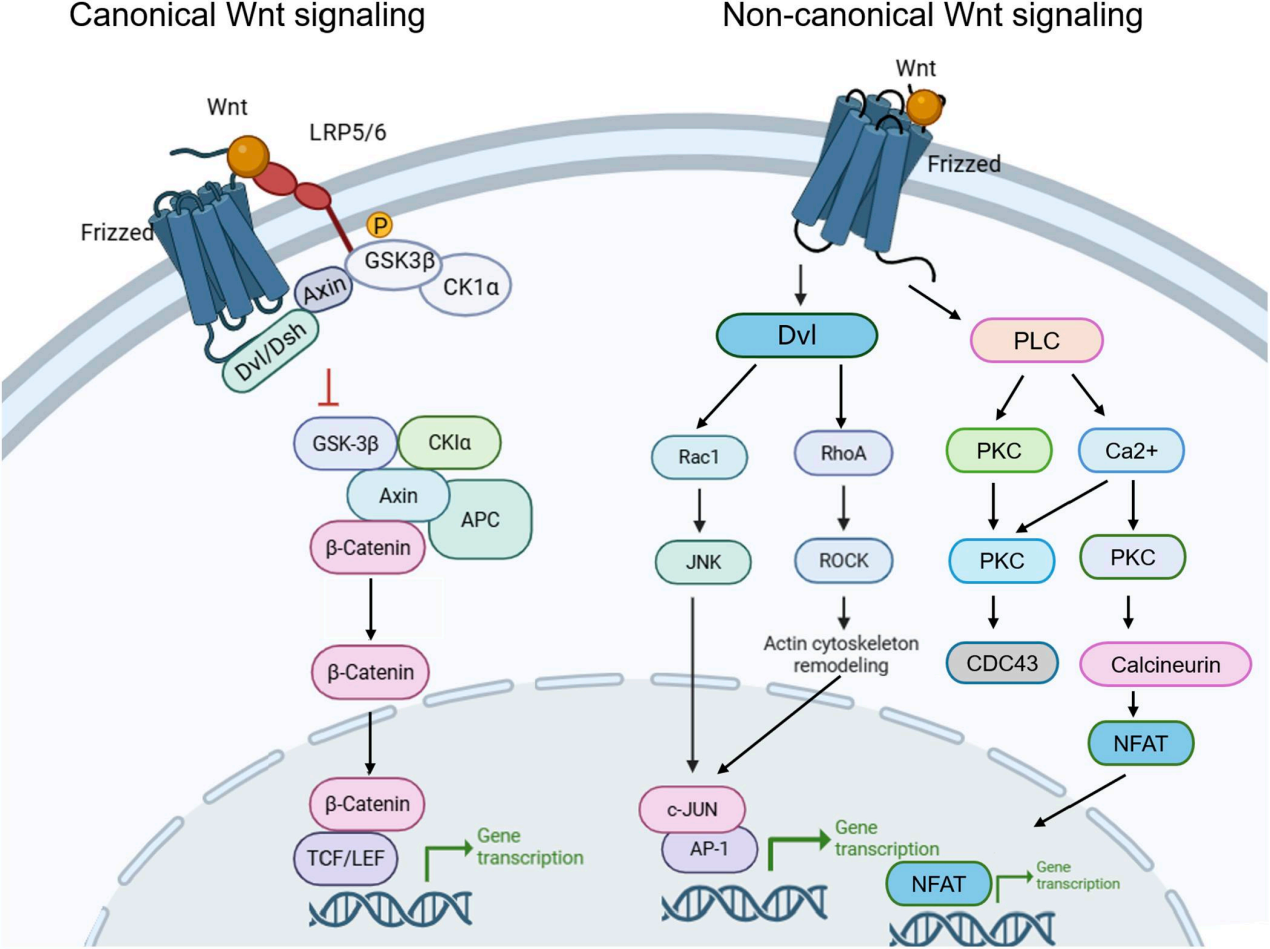
Damage to the Wnt signaling pathway regulates the progression of osteoarthritis. The classical and non-classical Wnt signaling pathways play a role in maintaining the stability of bone joints and influence bone formation.

Non-canonical Wnt signaling encompasses a group of β-catenin-independent pathways, including the Wnt/planar cell polarity (PCP) and Wnt/Ca^²⁺^ cascades, which are involved in the regulation of cytoskeletal organization, cell polarity, and chondrocyte differentiation (18). Wnt16, which displays only limited canonical signaling activity in chondrocytes, has been reported to exert a protective effect in OA. Experimental studies have shown that Wnt16 upregulates lubricin (PRG4) expression and protects cartilage from degeneration following medial meniscus destabilization (19). Mechanistically, Wnt16 appears to attenuate OA progression by suppressing cartilage catabolism and inhibiting chondrocyte hypertrophy through the PCP/c-Jun N-terminal kinas (JNK)–mammalian target of rapamycin complex 1 (mTORC1)–parathyroid hormone-related protein (PTHrP) signaling pathway (20, 21). Other non-canonical Wnt ligands, including Wnt5a and Wnt11, are also expressed in human OA cartilage and have been implicated in disease progression, although their specific roles in OA pathogenesis have yet to be fully clarified (22). Overall, both the canonical and non-canonical Wnt pathways are critically involved in the regulation of articular cartilage biology and in the pathogenesis of OA (Figure 2).

### 3.2 NF-κB signaling pathway

NF-κB is a family of transcription factors that plays a central role in the regulation of inflammation, cell differentiation, proliferation, and survival in mammalian cells (23). The canonical NF-κB pathway is triggered by extracellular stimuli such as TNF-α, IL-1β, or lipopolysaccharide (LPS), which bind to receptors such as toll-like receptors (TLRs) and initiate activation of the IκB kinase (IKK) complex. This complex phosphorylates IκB proteins, marking them for degradation and thereby permitting the p50/p65 dimer to translocate into the nucleus, where it promotes the transcription of proinflammatory cytokines and chemokines involved in coordinating immune responses. By contrast, the non-canonical NF-κB pathway is activated by a more restricted group of receptors, including CD40, receptor activator of NF-κB (RANK), and B-cell activating factor receptor, which stabilize NF-κB-inducing kinase and subsequently activate IKKα homodimers. These kinases mediate the processing of the p100 precursor into p52, resulting in the formation of p52/RelB complexes that regulate genes involved in lymphoid organogenesis and B-cell maturation. This pathway is generally slower in onset but more sustained than the canonical pathway.

NF-κB signaling plays important roles in cartilage development and endochondral ossification. Differential expression of NF-κB family members has been observed at distinct stages of cartilage maturation, with p65 detected throughout the growth plate and exhibiting particularly high expression in hypertrophic chondrocytes in both *in vitro* and *in vivo* settings, thereby suggesting a role in chondrocyte differentiation (24). Using tissue-specific knockout mouse models, Kobayashi et al. demonstrated that embryonic deletion of RelA/p65 in limb cartilage results in chondrocyte apoptosis and causes skeletal growth defects (25).

In OA, NF-κB signaling serves as a central mediator of pathological changes in articular cartilage. Genetic evidence supports an important role for the NF-κB pathway in OA progression. Variants in the *NFKBIA* gene, including rs8904, have been associated with increased inflammatory markers in female patients with OA, whereas functional single-nucleotide polymorphisms located within NF-κB binding motifs appear to modulate OA susceptibility in a sex-specific manner. Specifically, rs73164856 has been shown to confer protection against severe OA in males by downregulating AKR1B15 expression, while rs545654 significantly increases the risk of severe OA in females by upregulating neuronal nitric oxide synthase expression. In adult mice, homozygous deletion of Rela in articular chondrocytes accelerates arthritis progression, whereas heterozygous deletion reduces catabolic gene expression and delays disease onset, indicating that precisely regulated NF-κB activity is essential for joint homeostasis. Moreover, activation of YAP in articular chondrocytes inhibits transforming growth factor-β-activated kinase 1 (TAK1) downstream signaling and attenuates NF-κB activity, suggesting that cross-talk between the Hippo-YAP and NF-κB pathways is critical for maintaining cartilage homeostasis by regulating extracellular matrix-degrading enzymes (30).

Chondrocytes express a variety of membrane receptors, including mechanoreceptors, cytokine receptors, tumor necrosis factor receptors, and TLRs (26). Activation of these receptors by mechanical stress, proinflammatory cytokines (e.g., TNF-α and IL-1β), damage-associated molecular patterns such as fibronectin fragments, or other stimuli initiates NF-κB signaling and functionally intersects with other pathways, including bone morphogenetic protein (BMP) and Wnt signaling (29). Moreover, activation of YAP in articular chondrocytes has been reported to inhibit TAK1 downstream signaling and attenuate NF-κB activity, suggesting that cross-talk between the Hippo-YAP and NF-κB pathways is essential for maintaining cartilage homeostasis by modulating extracellular matrix-degrading enzymes (30). Canonical NF-κB activation induces the expression of multiple matrix-degrading enzymes, including MMP-1, -2, -3, -7, -8, -9, and -13, as well as aggrecanases such as a disintegrin and metalloproteinase with thrombospondin motifs (ADAMTS)-4 and -5, thereby promoting extracellular matrix degradation and articular cartilage erosion (27). NF-κB also regulates a broad array of cytokines and chemokines in OA chondrocytes, including TNF-α, IL-1β, IL-6, and RANK. These mediators further promote MMP production, inhibit the synthesis of type II collagen and proteoglycans, and amplify NF-κB activation through positive feedback mechanisms (28).

**Figure 3:**
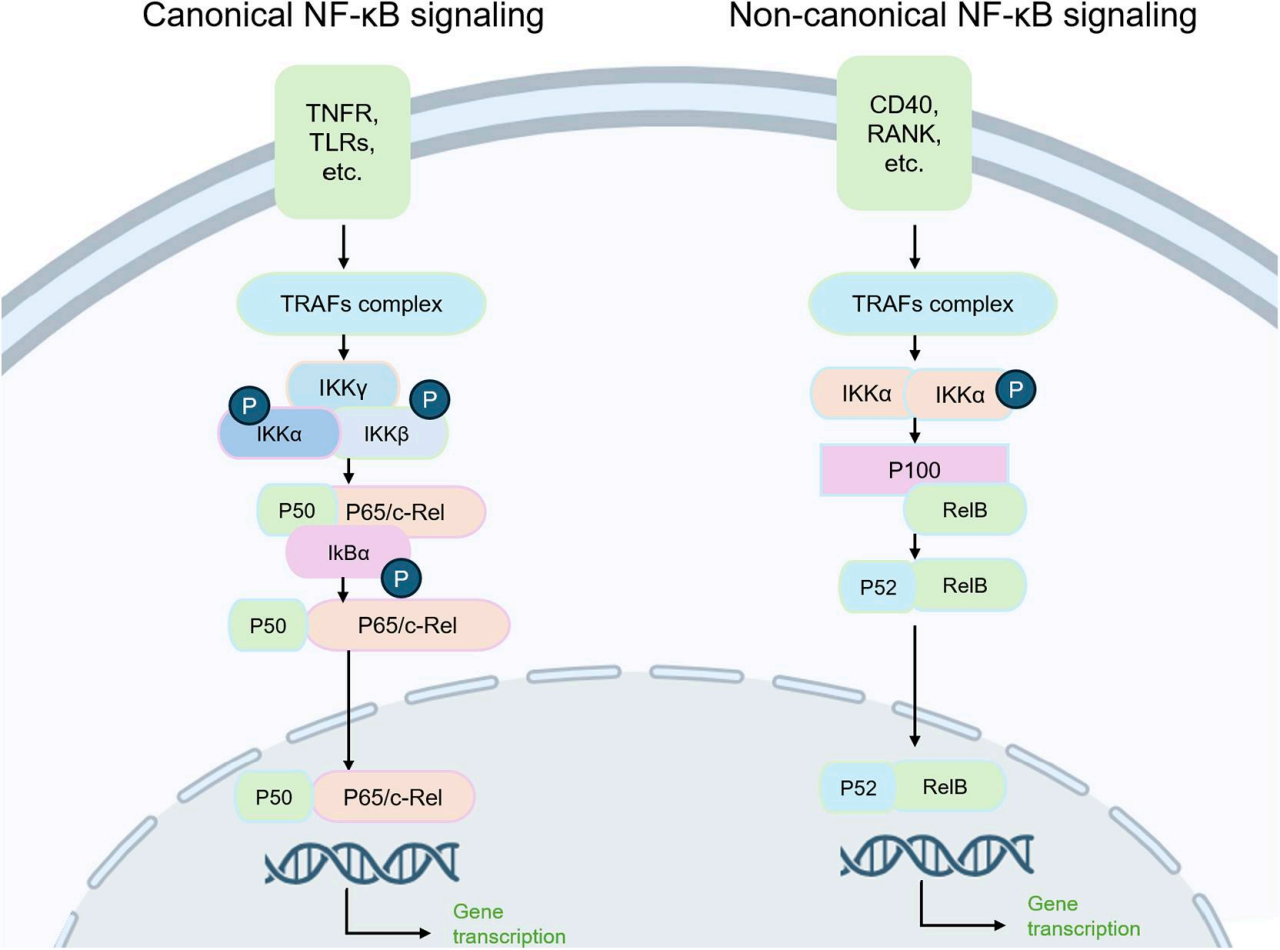
Damage to the NFκB signaling pathway is associated with the progression of OA. Damage to the classical NFκB and non-classical NKκB signaling pathways is associated with the local inflammatory progression of the knee joint, the apoptosis of chondrocytes, the reduction of matrix, and the damage of cartilage. It is also widely involved in the pathological progression of OA.

Moreover, NF-κB contributes to the increased expression of proinflammatory and catabolic mediators, including prostaglandin E2 (PGE2), NOS, and cyclooxygenase-2 (COX-2), which further aggravate synovial inflammation and chondrocyte death (Figure 3).

### 3.3 PI3K/AKT/mTOR signaling pathway

The PI3K/AKT/mTOR signaling pathway is a central regulator of cell growth, metabolism, proliferation, and survival, and its dysregulation has been linked to cancer, metabolic disorders, and neurodegeneration (31). The pathway is initiated when extracellular stimuli such as growth factors bind to receptor tyrosine kinases or G protein-coupled receptors (GPCRs), leading to the activation of phosphoinositide 3-kinase (PI3K), a p85/p110 heterodimer that converts phosphatidylinositol 4,5-bisphosphate into phosphatidylinositol 3,4,5-trisphosphate (PIP3) at the plasma membrane (32). This process is antagonized by phosphatase and tensin homolog. Accumulated PIP3 recruits both 3-phosphoinositide-dependent protein kinase 1 (PDK1) and AKT, enabling PDK1 to phosphorylate AKT at Thr308, whereas full AKT activation requires an additional phosphorylation at Ser473 by mTORC2 (33). Activated AKT then translocates to multiple subcellular compartments to regulate substrates involved in cell-cycle progression, apoptosis, and metabolism. A major downstream effector of AKT is mTOR, which forms two distinct complexes: mTORC1, which promotes protein synthesis and cell growth by phosphorylating ribosomal protein S6 kinase beta-1 (S6K1) and eukaryotic translation initiation factor 4E-binding protein 1 while inhibiting autophagy by suppressing unc-51-like kinase 1 (ULK1); and mTORC2, which regulates cytoskeletal organization and feeds back to enhance AKT activation (34). AKT activates mTORC1 by phosphorylating and inhibiting the tuberous sclerosis complex (TSC1/TSC2), which normally suppresses Ras homolog enriched in brain (Rheb), an upstream activator of mTORC1 (35). Through these mechanisms, the PI3K/AKT/mTOR axis exerts broad control over cell fate decisions by balancing anabolic activity with catabolic autophagy.

Accumulating evidence suggests that the PI3K/AKT/mTOR pathway is involved in osteoarthritis pathogenesis (36). Several studies have reported reduced PI3K/AKT signaling in human OA cartilage relative to normal cartilage, indicating that impaired activation of this pathway may contribute to disease-associated changes in chondrocyte function (40). The PI3K/AKT/mTOR pathway appears to participate in both ECM catabolic and anabolic processes. For instance, Venkatesan et al. demonstrated that MMP-13 expression in human OA chondrocytes and cartilage explants was reduced following rAAV-mediated overexpression of TGF-β, thereby implicating PI3K/AKT-dependent mechanisms in the suppression of catabolic enzymes (41). In another study, it was found that insulin-like growth factor-1 (IGF-1) promoted Col2a1 (type II collagen) expression and suppressed MMP-13 expression by activating PI3K and extracellular signal-regulated kinase (ERK) signaling (42). Pharmacological inhibition of PI3K with wortmannin significantly attenuated the effect of IGF-1 on Col2a1 expression but did not alter its effect on MMP-13, suggesting that distinct signaling branches downstream of IGF-1 differentially regulate anabolic and catabolic gene expression (37, 39). In contrast, leptin has been reported to upregulate MMP expression in chondrocytes by modulating PI3K/AKT signaling, further illustrating the context-dependent nature of this pathway in cartilage (43).

**Figure 4:**
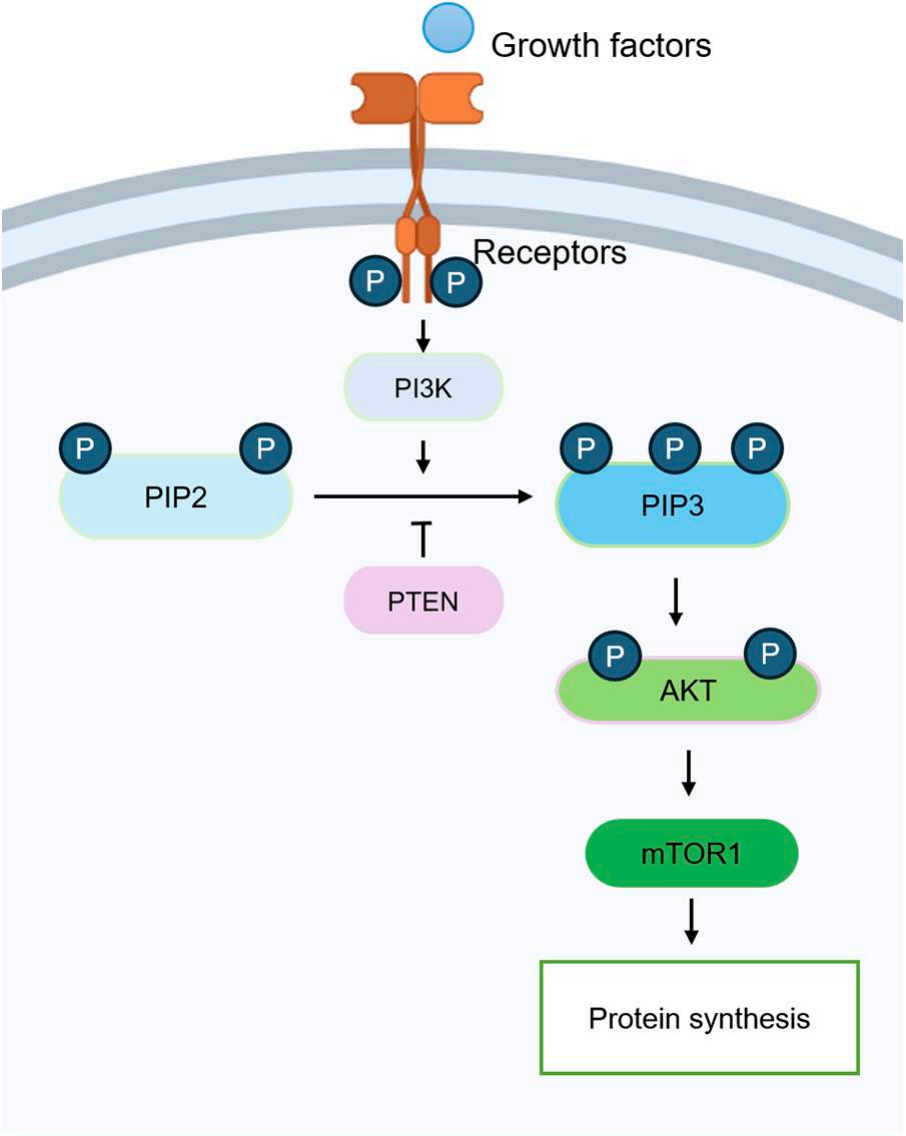
The PI3K/AKT/mTORC1 signaling pathway is involved in the pathogenesis of OA. The activation of the PI3K/AKT signaling pathway promotes the synthesis of the ECM and causes damage to the signal, which is involved in the progression of OA.

In summary, activation of the PI3K/AKT/mTOR pathway may promote ECM anabolism and chondrocyte survival; however, its precise role in matrix degradation and OA progression remains incompletely defined (44). Further investigation is required to clarify how PI3K/AKT/mTOR signaling contributes to cartilage homeostasis and to determine whether targeted modulation of this pathway could serve as a viable therapeutic strategy for OA (Figure 4) (38). 

### 3.4 TGF-β signaling pathway

The TGF-β superfamily, which includes TGF-β isoforms, BMPs, and growth differentiation factors, plays essential roles in skeletal development and cartilage homeostasis. Signaling is mediated through heteromeric complexes composed of type I and type II serine/threonine kinase receptors. To date, seven type I receptors and five type II receptors have been identified, and their signaling activity is often regulated by type III coreceptors. In the canonical pathway, ligand binding activates these receptor complexes, leading to the phosphorylation of receptor-regulated SMADs (R-SMADs): BMP receptors primarily activate SMAD1/5/8, whereas TGF-β receptors preferentially activate SMAD2/3 (45, 46). These phosphorylated R-SMADs subsequently form complexes with the common mediator SMAD4, translocate to the nucleus, and regulate target gene transcription in cooperation with other transcriptional partners. In addition, TGF-β/BMP receptors can activate non-canonical, SMAD-independent pathways, including TAK1 and extracellular signal-regulated kinase 1/2 (ERK1/2), thereby further broadening the range of cellular responses to these ligands (47).

In articular cartilage, TGF-β is produced by chondrocytes and plays an important role in preserving tissue integrity, particularly under conditions of excessive mechanical loading, as shown in both *in vitro* and *in vivo* OA models (51). Activation of TGF-β/SMAD2/3 signaling in articular chondrocytes promotes the expression of extracellular matrix components, including type II collagen and fibronectin, while concurrently suppressing catabolic responses triggered by injurious mechanical stress (52). TGF-β has also been reported to inhibit MMP-13 expression, in part by inducing anti-inflammatory mediators, and to suppress cartilage matrix degradation by upregulating SOX9, a master transcription factor of chondrogenesis (53). The TGF-β-responsive gene 2,3′-phosphoadenosine 5′-phosphosulfate synthetase 2 is required for physiological proteoglycan sulfation and thus contributes to appropriate extracellular matrix assembly (50). Boyan et al. demonstrated that the activation of TGF-β signaling by 25-hydroxyvitamin D3 inhibited IL-1β-mediated inflammatory signaling, reduced chondrocyte apoptosis, and suppressed the expression of matrix-degrading enzymes (54). In addition, Prg4, the gene encoding lubricin, is regulated by TGF-β, and its product enhances boundary lubrication at the articular surface, thereby protecting cartilage from wear-related damage (55).

**Figure 5:**
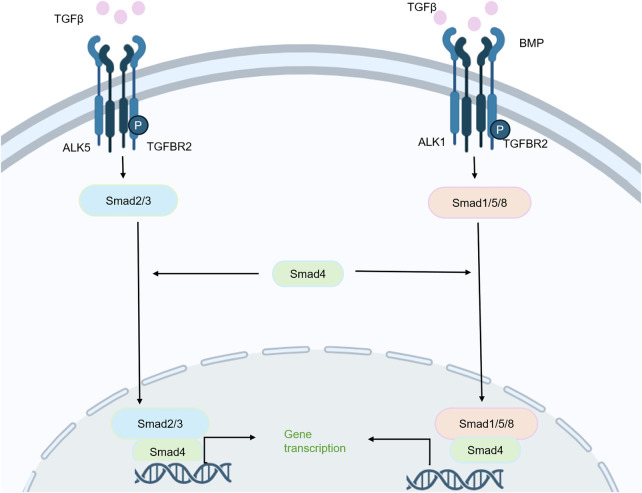
The TGFβ signaling pathway is involved in the pathogenesis of OA. Both the TGFβ/Smad2/3 and BMP/Smad1/5/8 signaling impairments contribute to the progression of OA by mediating matrix production and cartilage formation.

TGF-β also exerts anti-osteoarthritic effects by regulating specific microRNAs (miRNAs) (48). For instance, TGF-β has been shown to suppress AMP-activated protein kinase and p38 mitogen-activated protein kinase signaling by modulating miR-92a, while simultaneously upregulating miR-135b and miR-140-5p to inhibit chondrocyte apoptosis and cartilage matrix degradation (Figure 5) (56). Despite these advances, the precise and potentially distinct contributions of TGFβ receptor type I and type II to the maintenance of articular cartilage homeostasis remain to be fully elucidated (57). Similarly, the regulatory mechanisms and biological significance of non-canonical BMP signaling in preserving cartilage homeostasis and in the initiation and progression of OA remain to be fully clarified (49).

## 4 Components of PRP

PRP is a biologically active autologous blood-derived concentrate composed predominantly of platelets, with variable amounts of leukocytes and erythrocytes also present. Each of these cellular components may influence tissue repair and regeneration through distinct mechanisms. Platelets are considered the primary mediators of the anabolic and regenerative properties of PRP, largely through the release of bioactive growth factors and cytokines. By contrast, leukocytes and erythrocytes exert modulatory effects on reparative processes that may be either beneficial or detrimental, depending on their concentration, activation state, and the biological characteristics of the target tissue.

### 4.1 Platelets in PRP

Thrombocytes, commonly known as platelets, are anucleate cell fragments generated through cytoplasmic budding from megakaryocytes in the bone marrow (58). Megakaryocytes extend long cytoplasmic processes through the sinusoidal endothelium, where platelets are released directly into the bloodstream (59). After entering the circulation, platelets continuously interact with the vascular endothelium and play essential roles in surveilling endothelial integrity and initiating hemostatic responses in the event of vascular injury.

PRP preparations typically contain platelet concentrations approximately 3–5 times greater than those in baseline whole blood, leading to a corresponding increase in intraplatelet growth factor content (60). Upon activation, platelets release a wide array of bioactive mediators from their α-granules, including TGF-β, platelet-derived growth factor (PDGF) isoforms, IGFs, vascular endothelial growth factor (VEGF), and epidermal growth factor. These mediators promote angiogenesis, tissue regeneration, and cellular proliferation within the local microenvironment (Table 1) (61). However, supraphysiological concentrations of certain factors may paradoxically reduce regenerative efficacy, highlighting the importance of optimizing PRP composition and dosing (62).

**Table 1:** Types of growth factors in PRP: cellular sources and functions.

PGF	Cellular sources	Functions
PDGF	Platelets, endothelial cells, macrophages, and smooth muscle cells	Stimulates chemotaxis and mitosis of fibroblasts, glial cells, and smooth muscle cells
TGF	Macrophages, T lymphocytes, and keratinocytes	Regulates mitosis in endothelial cells, fibroblasts, and osteoblasts
VEGF	Platelets, macrophages, keratinocytes, and endothelial cells	Promotes angiogenesis and vascular permeability
EGF	Platelets, macrophages, and monocytes	Stimulates keratinocyte proliferation and fibroblast proliferation
a-bFGF	Platelets, macrophages, mesenchymal cells, chondrocytes, and osteoblasts	Promotes growth and differentiation of chondrocytes and osteoblasts
CTGF	Platelets and fibroblasts	Promotes angiogenesis, cartilage regeneration, and fibrosis
IGF-1	Platelets, plasma, epithelial cells, endothelial cells, fibroblasts, osteoblasts, and bone matrix	Promotes fibroblast chemotaxis and protein synthesis to enhance osteoblast proliferation
HGF	Platelets and mesenchymal cells	Regulates epithelial/endothelial cell growth and motility to support repair and angiogenesis
KGF	Fibroblasts and mesenchymal cells	Regulates epithelial cell migration and proliferation
Ang-1	Platelets and neutrophils	Induces angiogenesis and stimulates endothelial cell migration and proliferation
PF4	Platelets	Activates white blood cells and regulates their activation to eliminate microbial activity
SDF-1α	Platelets, endothelial cells, and fibroblasts	Becomes CD34+ cells, inducing their homing and proliferation
TNF	Macrophages, mast cells, and T lymphocytes	Regulates monocyte migration, fibroblast proliferation, macrophage activation, and angiogenesis

In addition to α-granules, platelets contain dense (δ) granules and lysosome-like (λ) granules, which harbor a wide range of small molecules, enzymes, and other bioactive mediators (63). These granule populations are generated during megakaryocyte maturation and are further shaped by endocytic uptake from the circulation (64). Collectively, their contents contribute to the regulation of key processes involved in tissue repair, including inflammatory modulation, progenitor cell recruitment and differentiation, and extracellular matrix remodeling.

### 4.2 The role of plasma in PRP

Blood plasma, the liquid component of blood, contains a broad array of biomolecules that collectively reflect the body's physiological and pathological status. The total plasma protein concentration typically ranges from 60 to 80 mg/mL. Albumin constitutes approximately 60% of total plasma protein, globulins about 30%, and fibrinogen around 4% (65). The remaining approximately 1% comprises several thousand low-abundance proteins, many of which are involved in critical signaling pathways that regulate tissue homeostasis and disease progression (66).

The targeted application of plasma-derived proteins has emerged as a promising strategy in regenerative medicine and tissue engineering, particularly for enhancing tissue repair and promoting musculoskeletal regeneration (67). Structural plasma proteins such as fibronectin, fibrin, and vitronectin are not only essential components of hemostasis but also facilitate stromal cell adhesion and migration, thereby supporting tissue repair and remodeling following injury (68). In addition, plasma functions as a carrier for numerous circulating hormones, including thyroxine, adrenocorticotropic hormone, androgens, estrogens, progesterone, and human growth hormone (hGH) (69). Among these, IGF-1 plays a particularly important role in skeletal muscle repair, and accumulating experimental evidence indicates that it also contributes to tendon and cartilage healing (70).

### 4.3 Leukocytes in PRP

Leukocytes play a central role in the regulation of inflammation, orchestration of host immune responses, and support of wound healing. Neutrophils are key effector cells that form during the early phase of tissue repair, rapidly accumulating at sites of injury to provide a first line of defense against invading pathogens (71). In addition to their antimicrobial functions, recent studies have shown that neutrophils may also contribute to neovascularization and tissue repair by releasing proangiogenic and proregenerative mediators (72). However, under certain conditions, persistent neutrophil activation and the release of inflammatory cytokines and MMPs can promote catabolic processes and contribute to tissue degradation (73).

**Figure 6:**
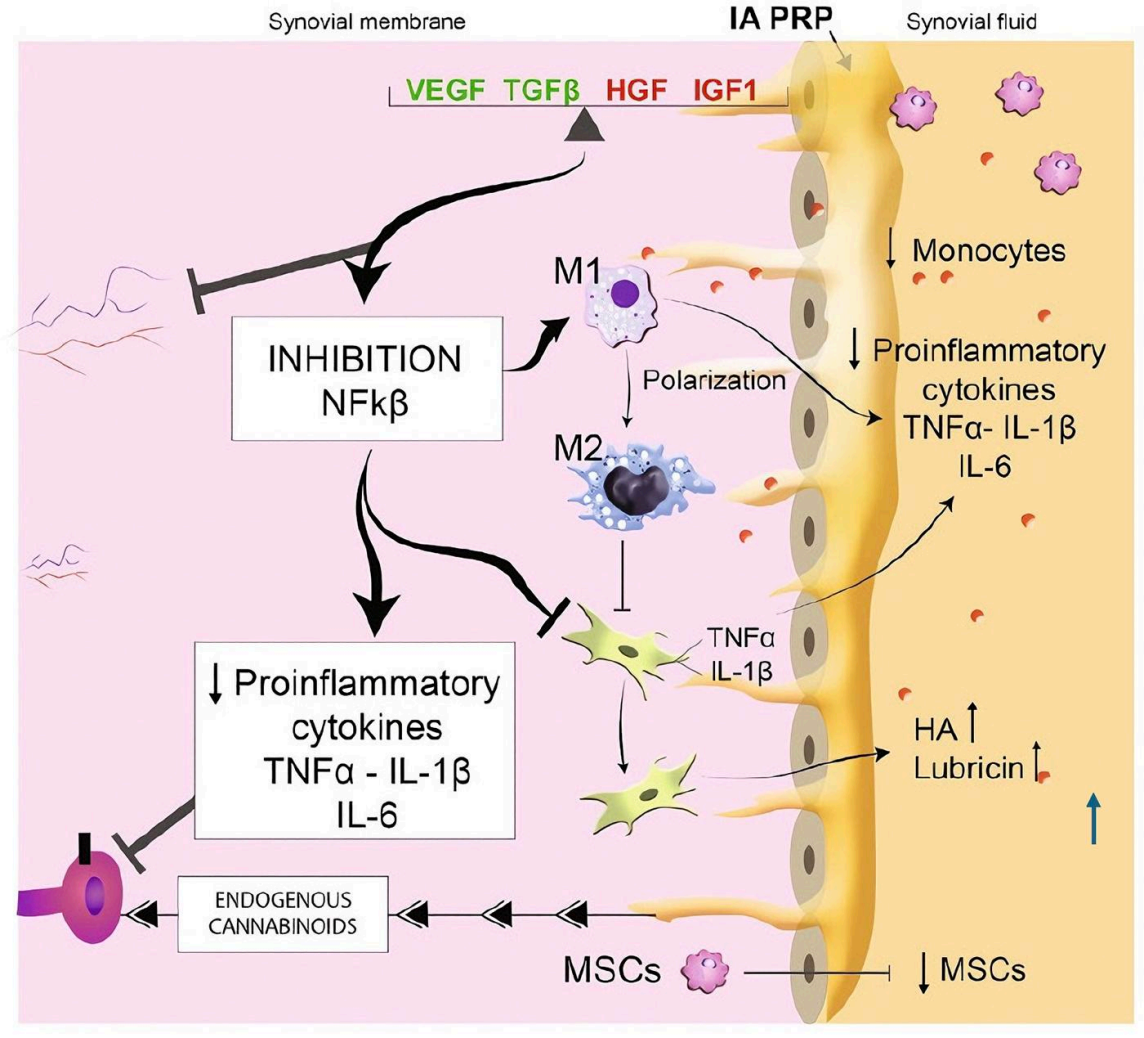
Mechanism of PRP in restoring homeostatic imbalance in osteoarthritis. PRP may disrupt the catabolic cycle and suppress inflammatory responses in osteoarthritis (OA) through its unique anti-inflammatory effects. The key pathological context of OA involves prolonged exposure of chondrocytes and synovial cells to proinflammatory cytokines, abnormal mechanical stress, and disease-associated molecular patterns. This leads to a chronic injury-like state within the joint—characterized by a disruption of extracellular matrix (ECM) homeostasis and early inflammatory responses. PRP specifically targets this pathological context.

The monocyte content of PRP varies depending on the blood collection and processing protocol, and the precise contribution of monocytes to PRP-mediated tissue repair remains to be fully defined (74). Monocytes are derived from hematopoietic stem cells in the bone marrow and circulate in peripheral blood until they are recruited by local chemoattractants to sites of tissue injury or inflammation. Upon entering the tissue, they differentiate into macrophages or dendritic cells and become part of the mononuclear phagocyte system (75). Macrophages exhibit considerable phenotypic plasticity and can assume distinct activation states in response to local environmental cues. Classically activated M1 macrophages produce proinflammatory cytokines, nitric oxide, and growth factors such as VEGF and fibroblast growth factor (FGF), thereby contributing to host defense and early angiogenic responses (76). By contrast, alternatively activated M2 macrophages, including the M2a, M2b, and M2c subtypes, exert anti-inflammatory and reparative functions by secreting IL-10, extracellular matrix components, and additional angiogenic mediators (77). Importantly, macrophages can switch between M1 and M2 phenotypes *in vivo* in a tightly regulated manner, with cytokines such as IL-4 playing key roles in this polarization process (78). Further studies are required to clarify how leukocyte subsets—particularly macrophages—within PRP preparations influence the balance between inflammation and repair in musculoskeletal tissues (Figure 6).

### 4.4 The effect of red blood cells (RBCs) in PRP

The preparation of PRP generally aims to minimize the content of RBCs, as these cells have limited regenerative potential and may exert cytotoxic effects (79). Under physiological conditions, RBCs are responsible for oxygen transport through hemoglobin. However, when RBCs undergo hemolysis, particularly in the setting of oxidative stress, they can release free iron and generate reactive oxygen species, which may induce cell death, including apoptosis, and contribute to local tissue injury (80). Accordingly, reducing RBC contamination in intra-articular PRP preparations is considered important for optimizing both the safety and the therapeutic efficacy of joint injections (81).

### 4.5 Factors influencing PRP in cartilage repair

A major challenge in the clinical application of PRP for OA, particularly knee OA, is the lack of standardization in the optimal dose, platelet concentration, and injection frequency (Table 2). Current clinical studies employ heterogeneous PRP preparation protocols and treatment regimens, which likely contribute to the variability observed in reported outcomes.

**Table 2:** Differences between single and multiple PRP injections.

Injection strategy	Theoretical rationale	Potential advantages	Potential disadvantages	Clinical pattern suggested
Single injection	Delivers one concentrated biologic stimulus with minimal procedural burden	Lower cost, fewer visits, reduced injection-related discomfort, and better convenience and adherence	May provide a shorter duration of benefit; may be insufficient for chronic or advanced disease	Evidence summarized in your manuscript indicates that single-dose PRP may yield better short-term improvement, whereas durability may be less robust
Multiple injections (commonly two to three injections, often 1–4 weeks apart)	Repeated biologic stimulation may sustain anti-inflammatory and anabolic signaling over time	Potentially stronger mid- to long-term symptom control; may improve cumulative therapeutic effect in chronic disease	Higher cost, more clinic visits, greater cumulative postinjection irritation, and more protocol heterogeneity	Superior efficacy with higher-dose regimens, particularly three injections, while your manuscript also notes that multiple injections tend to provide more pronounced long-term benefit

With respect to platelet content, the clinical literature commonly recommends PRP platelet concentrations approximately four to six times greater than those in whole blood. However, concentrations exceeding the optimal range may confer no additional therapeutic benefit and may even inhibit tissue repair. *In vitro* studies have demonstrated that a concentration of 1.5 × 10^6^ platelets/µL is optimal for promoting the proliferation, motility, and morphology of human umbilical vein endothelial cells, whereas higher concentrations may produce inhibitory effects (130). In normal human dermal fibroblasts, a platelet concentration of 0.5 × 10^6^ to 1.5 × 10^6^ platelets/µL has been reported to be optimal for promoting cell proliferation, motility, and wound healing (131). Likewise, tenocyte function increases with rising platelet concentration but plateaus at approximately 2.0 × 10^6^ platelets/µL, beyond which no additional functional benefit is observed (132).

PRP activation further contributes to variability in growth factor release. Activation has been shown to modify the platelet concentration required to achieve the proliferation plateau in tenocytes, underscoring that the biological activity of PRP depends not only on platelet concentration but also on activation status (133). Both thrombin and calcium exhibit dose-dependent effects on platelet-derived growth factor release: higher concentrations trigger a more rapid and substantially greater release of anabolic growth factors, whereas lower concentrations lead to delayed and reduced release (134). In comparison with thrombin activation, collagen activation results in a more sustained release profile. Although activation can accelerate growth factor release, this rapid release may not necessarily optimize therapeutic efficacy (135). For example, thrombin-activated PRP has been shown to be less effective for wound healing than non-activated PRP.

The optimal number of PRP injections remains a matter of debate. Available evidence suggests that single-injection and multiple-injection protocols may yield different therapeutic outcomes in the treatment of osteoarthritis (136). For instance, a meta-analysis assessing the efficacy of PRP for osteoarthritis, which included 70 studies involving 2,530 patients, reported that a single PRP injection was associated with greater short-term clinical improvement, whereas multiple injections conferred more pronounced benefits over longer-term follow-up (129).

Accordingly, PRP treatment protocols should be individualized based on patient tolerance, symptom severity, and therapeutic goals. For patients with severe or persistent symptoms, or for those who have responded inadequately to other conservative treatments, multiple PRP injections in combination with a structured rehabilitation program may represent a more appropriate strategy. By contrast, a single PRP injection may be considered for patients with milder symptoms who primarily seek short-term pain relief, although this approach may be less suitable for achieving durable long-term outcomes.

### 4.6 PRP preparation methods

The primary objective of PRP preparation technology is to achieve efficient separation and concentration of platelets while preserving their biological activity. Standardized PRP preparation is essential to ensure consistent quality in both basic research and clinical applications (137). Nevertheless, universally accepted standards for PRP preparation remain unavailable (Table 3).

**Table 3:** PRP formulations, theoretical advantages, potential risks, and current evidence strength.

PRP category	Theoretical advantages	Potential risks	Current strength of evidence
Leukocyte-poor PRP (LP-PRP)	Lower leukocyte burden may reduce intra-articular inflammation, synovial irritation, and catabolic cytokine exposure; often considered more suitable for intra-articular use	May provide less innate immune stimulation; preparation methods remain heterogeneous	Moderate for symptomatic knee OA, especially short- to mid-term improvement; frequently favored in reviews because of a more favorable inflammatory profile, although direct superiority over LR-PRP is not consistently proven
Leukocyte-rich PRP (LR-PRP)	Higher leukocyte content may enhance early immunomodulation, antimicrobial activity, and release of certain bioactive mediators; may be theoretically useful where a stronger early inflammatory/healing response is desirable	Higher risk of postinjection pain, swelling, and reactive synovitis; excessive inflammatory signaling may be counterproductive in joint disease	Moderate-to-low, depending on indication. In knee OA, an RCT found similar clinical improvement vs. LP-PRP, with numerically more mild adverse events in LR-PRP
Activated PRP (e.g., calcium/thrombin-activated)	Immediate platelet degranulation and rapid growth factor release; may be advantageous when a prompt anabolic signal or gel/clot formation is desired	Rapid release kinetics may shorten biologic persistence; activation method affects product composition; thrombin-based activation may alter biologic behavior and increase protocol variability	Low for clear clinical superiority over non-activated PRP in routine musculoskeletal injection therapy; current evidence remains inconsistent and highly protocol-dependent
Non-activated PRP	May provide a more gradual, physiologic release of growth factors after exposure to endogenous collagen and tissue signals; simpler protocol with less manipulation	Slower onset of mediator release; efficacy may depend more strongly on tissue microenvironment	Moderate as a practical clinical approach, but evidence is mainly indirect; superiority over activated PRP has not been firmly established across indications
High-platelet-concentration PRP	Greater platelet payload may increase concentrations of PDGF, TGF-β, and other anabolic mediators; may improve symptom response when the concentration is within a biologically effective range	Excessively high concentrations may show diminishing returns or even inhibitory effects; not all “high-dose” products are biologically equivalent	Moderate for an association with better outcomes in knee OA, but the optimal concentration window remains uncertain
			Some study reported better outcomes and lower failure rates with higher platelet concentration
Low-platelet-concentration PRP	Potentially lower inflammatory reactivity and easier preparation in some systems	May be biologically subtherapeutic; lower growth factor delivery may reduce efficacy	Available evidence suggests inferior performance compared with more concentrated preparations in knee OA
PRP-derived exosomes	Cell-free biologic strategy; may preserve paracrine regenerative signaling while reducing cellular variability; theoretically attractive for cartilage regeneration and precision biologics	Manufacturing, purification, dosing, regulatory classification, and long-term safety are unresolved; currently not standardized for routine clinical use	Very low/exploratory; evidence is predominantly preclinical, and robust human clinical validation is lacking

PRP preparation techniques can be classified according to their underlying methodological principles, with density gradient centrifugation and membrane filtration representing the most commonly used approaches (137). Density gradient centrifugation separates blood components based on differences in their sedimentation behavior under centrifugal force, thereby producing stratification of cellular elements. By contrast, membrane filtration isolates particles of specific sizes, such as platelets, using selective filtration membranes (Table 4).

**Table 4:** Sedimentation coefficients of blood components.

Blood components	Plasma	Platelets	Lymphocytes	Monocytes	Neutrophils	Red blood cells	Whole blood
Settlement coefficient	1.027	1.040	1.055	1.065	1.090	1.096	1.050

At present, density gradient centrifugation remains the most widely used method for clinical PRP preparation owing to its relative simplicity, efficiency, and capacity for effective platelet enrichment.

Density gradient centrifugation is a commonly used technique for isolating and extracting PRP from whole blood by taking advantage of differences in the sedimentation behavior of blood components under centrifugal force (138). The platelet concentration and biological activity of the final PRP product are influenced by several preparation parameters, including centrifugal force, the number of centrifugation cycles, centrifugation time, and temperature conditions during processing.

In human whole blood, platelets are approximately 2–3 µm in diameter, red blood cells average about 9 µm, and white blood cells range from 7 to 20 µm in diameter. Membrane filtration utilizes the sieving properties of membranes with defined pore sizes to separate blood components of different sizes, such as red blood cells and leukocytes, from platelets in whole blood (139).

For example, the primary layer of a large-pore membrane retains red blood cells and leukocytes, whereas the secondary layer of a small-pore membrane selectively captures platelets. After elution, the collected fraction yields PRP that meets the desired specifications. In addition, membrane filtration can be combined with centrifugation to further enhance the purity and efficiency of PRP preparation.

### 4.7 The role of leukocyte concentration in therapeutic efficacy

The relative advantages of leukocyte-poor PRP (LP-PRP) and leukocyte-rich PRP (LR-PRP) have been extensively investigated, given that leukocytes may exert proinflammatory effects and potentially hinder tissue repair. Interactions between mononuclear cells and platelets can enhance the release of anabolic growth factors and stimulate cellular activity (140). However, excessively high leukocyte concentrations (>21,000 µL), particularly neutrophil-rich preparations, are associated with increased release of proinflammatory and catabolic mediators, regardless of the platelet-to-leukocyte ratio (141). Such responses may trigger acute inflammation and increase synoviocyte death. Accordingly, LP-PRP (<1,000 µL) is often regarded as a more favorable option. Nevertheless, the optimal leukocyte concentration and the ideal leukocyte-to-platelet ratio in PRP remain to be determined. Most PRP preparation methods also seek to minimize red blood cell content, typically reducing concentrations to below 1,000 µL (142).

The composition and quality of PRP preparations generated by different preparation methods remain incompletely characterized. In particular, the role of leukocytes in PRP formulations remains controversial, and relatively few studies have systematically investigated how platelet–leukocyte interactions affect growth factor concentrations, proinflammatory activity, and cellular responses (143).

A substantial body of evidence, primarily from orthopedics and sports medicine, supports the use of LP-PRP. By contrast, LR-PRP has also been reported to contribute to several biological processes relevant to tissue repair, including angiogenesis and extracellular matrix remodeling (144). For instance, Kobayashi et al. observed a positive correlation between leukocyte concentration and the levels of PDGF-BB and VEGF, whereas FGF-β levels were negatively correlated with leukocyte concentration (145).

Similarly, in a rabbit model of chronic tendon pathology, Yan et al. reported that collagen fiber diameter was significantly greater in the LP-PRP-treated group than in the LR-PRP group (146). However, both PRP-treated groups exhibited significantly lower expression of MMP-1 and MMP-3 compared with the control group, suggesting a potential inhibitory effect on matrix degradation (147). LR-PRP appeared to produce stronger chemotactic and proliferative effects, whereas LP-PRP was associated with higher rates of cell migration within the tendon lesion. Notably, LR-PRP also elicited a greater proinflammatory response, as indicated by increased IL-6 secretion.

Given the differences in formulation characteristics and biological effects between LP-PRP and LR-PRP, the choice of PRP formulation should be guided by the temporal requirements and biological characteristics of the injured tendon. Consequently, the development of tailored PRP protocols will likely depend on multifunctional preparation systems capable of producing customized PRP compositions.

## 5 Mechanisms of signaling regulation in PRP

**Figure 7:**
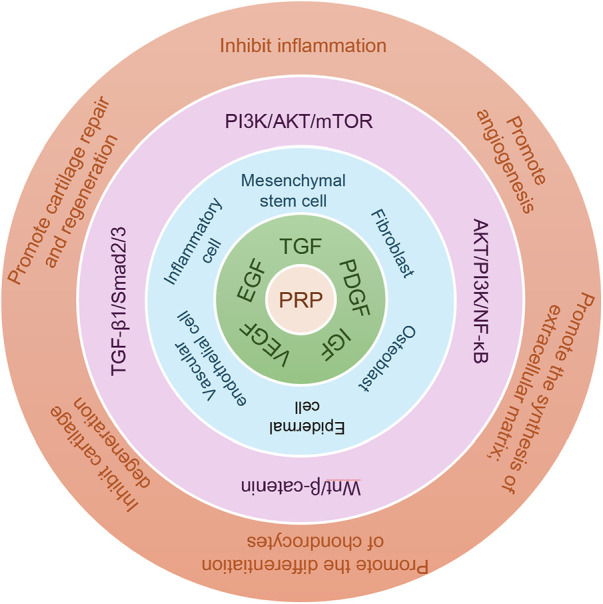
Mechanisms of signaling regulation in PRP. When platelets are activated, they can release a large amount of growth factors. When PRP is activated, it can release five to eight types of growth factors that accelerate wound healing, such as the following: transforming growth factor TG, platelet-derived growth factor PDGF, insulin-like growth factor IGF, vascular endothelial cell growth factor VEGF, epidermal growth factor EGF, FGF, and so on, which act on mesenchymal stem cells, osteoblasts, fibroblasts, vascular endothelial cells, and epidermal cells. The mechanism involves the PI3K/AKT/mTOR, NF-κB, Wnt/β-catenin, and TGF-β/Smad signaling pathways, achieving the effects of inhibiting inflammation, promoting angiogenesis, extracellular matrix synthesis, and chondrocyte differentiation, inhibiting chondral degeneration, and also promoting chondral repair and regeneration.

The principal mechanism by which PRP exerts therapeutic effects in joint disease is its capacity to modulate key signaling pathways in articular chondrocytes and surrounding tissues. Through such regulation, PRP may influence inflammatory responses, autophagy, extracellular matrix metabolism, and cell survival. OA progression is closely linked to dysregulation of pathways such as PI3K/AKT/mTOR, NF-κB, Wnt/β-catenin, and TGF-β/Smad. In the following section, we summarize current evidence regarding how PRP and PRP-derived factors alter the chondrocyte microenvironment by inhibiting, activating, or fine-tuning these signaling networks. Such effects may contribute to delaying cartilage degeneration and promoting tissue repair and regeneration. A clearer understanding of these regulatory mechanisms will be essential for optimizing PRP formulations and enhancing their clinical efficacy (Figure 7).

## 6 PRP inhibits the PI3K/AKT/mTOR signaling pathway

In an animal study conducted by Cheng et al., it was found that intra-articular administration of PRP significantly improved cartilage structure, reduced Mankin scores, and altered the mRNA expression of proinflammatory cytokines, including IL-1β, IL-6, IL-18, and TNF-α, as well as the catabolic enzyme MMP-13, in cartilage tissue, indicating substantial anti-inflammatory and antidegradative effects (82). Additional experimental studies have shown that pharmacological inhibition of the PI3K/AKT/mTOR pathway induces autophagy in chondrocytes, as evidenced by an increased expression of autophagy-related genes such as ATG5, Beclin-1, and ULK1, an increased LC3-II/LC3-I ratio, and reduced levels of the autophagy substrate p62; these changes are similar to those observed with the mTOR inhibitor rapamycin. In contrast, these effects are reversed by the mTOR activator MHY1485, underscoring the central role of PI3K/AKT/mTOR signaling in autophagy regulation. On the basis of these findings, PRP has been proposed to enhance chondrocyte autophagy, at least in part, through attenuation of PI3K/AKT/mTOR signaling, thereby reducing inflammation, slowing cartilage degradation, and supporting tissue repair. Nevertheless, this hypothesis requires further confirmation in well-designed preclinical and clinical studies (Table 5).

**Table 5:** Studies on the role of the PI3K/AKT/mTOR pathway in PRP-related applications.

References	Model type (animal/human)	*In vivo*/*in vitro*	Study type	Type of cartilage injury	Model of injury (natural vs. induced)
Zhang et al. (83)	Animal ACL reconstruction model	*In vivo*	Preclinical (animal study)	Primarily tendon-bone interface healing, possibly accompanied by localized cartilage/interface damage	Induction (surgical ACL reconstruction+graft implantation)
Xu, (85)	Rat knee OA model + PRP	*In vivo*	Preclinical (animal study)	OA-like degenerative articular cartilage damage	Induction

## 7 PRP inhibits AKT/PI3K/NF-κB activation

Preclinical studies suggest that PRP exerts protective effects on articular cartilage under various injurious conditions. In a doxorubicin-induced cartilage injury model, intra-articular PRP reduced chondrocyte apoptosis and matrix degradation by inhibiting NF-κB activation and decreasing downstream proinflammatory mediators, including inducible NOS, TNF-α, and interleukins (83). In other experimental models, PRP administration promoted chondrogenesis within repair tissue at the defect site, with substantial cartilage formation observed a later time point, and was associated with suppression of AKT/PI3K/NF-κB signaling and attenuation of inflammatory responses (84).

Multiple cytokines present in PRP appear to contribute to its anti-inflammatory effects by modulating NF-κB activity (119). Hepatocyte growth factor and TNF-α have been reported to negatively regulate NF-κB-dependent transcription and to suppress the expression of NF-κB target genes, including COX-2 and the chemokine receptor CXCR4 (85). TGF-β1 can antagonize TNF-α-induced chemokine transactivation, thereby inhibiting monocyte chemotaxis. In addition, activated PRP has been shown to reduce the expression of CXCR4 and NF-κB-regulated chemokines such as monocyte chemoattractant protein-1 and RANTES in monocyte-like cells, providing a potential mechanistic basis for its inhibitory effects on inflammatory cell recruitment (86). Collectively, these findings suggest that the balance between proinflammatory and anti-inflammatory mediators within PRP is a critical determinant of its net effect on NF-κB signaling and joint inflammation.

Importantly, the leukocyte content of PRP can significantly influence NF-κB activation and downstream responses. Wenjing Yin and colleagues reported that L-PRP robustly activated NF-κB signaling in human articular chondrocytes, in contrast to pure-PRP, which showed less pronounced NF-κB activation (87). Although L-PRP supported extracellular matrix synthesis, the presence of leukocytes increased the levels of IL-1β and TNF-α, thereby stimulating NF-κB and potentially counteracting some of the anabolic effects of platelet-derived growth factors on human articular chondrocytes. These observations underscore the importance of distinguishing between leukocyte-rich and LP-PRP formulations and suggest that excessive leukocyte content may diminish the net regenerative benefit, at least *in vitro*. Further *in vivo* studies are needed to clarify how different PRP compositions—particularly with respect to leukocyte concentration—affect NF-κB signaling, inflammation, and cartilage repair in osteoarthritis, and to identify formulations that maximize clinical benefit (Table 6) (120).

**Table 6:** Studies on the role of the AKT/PI3K/NF-κB pathway in PRP-related applications.

References	Model type (animal/human)	*In vivo*/*in vitro*	Study type	Type of cartilage injury	Model of injury (natural vs. induced)
Zhang et al. (83)	Animal ACL reconstruction model	*In vivo*	Preclinical (animal study)	Primarily tendon-bone interface healing, possibly accompanied by localized cartilage/interface damage	Induction (surgical ACL reconstruction + graft implantation)
Zhao et al. (82)	Articular chondrocytes	*In vitro*	Basic research	Adriamycin-induced inflammatory cartilage injury	Induction (adriamycin drug treatment)
Bendinelli et al. (84)	Human knee articular chondrocytes	*In vitro*	Basic research	Degenerative joint cartilage disease (OA-related)	Occurred naturally (surgical specimens from patients with knee osteoarthritis)
Yin et al. (86)	Human articular chondrocytes (comparison of multiple PRP formulations, some sourced from knee OA)	*In vitro*	Basic research	OA-related degenerative cartilage damage (selected samples)	Occurred naturally (OA surgery specimen)
Xu et al. (85)	Rat knee osteoarthritis model	*In vivo*	Preclinical (animal study)	OA degenerative joint cartilage damage	Induction

## 8 PRP and exosomes in Wnt/β-catenin signaling

Recent evidence suggests that both PRP and PRP-derived exosomes (PRP-Exos) play important roles in modulating OA progression, largely by regulating the canonical Wnt/β-catenin signaling pathway in inflammatory models of cartilage damage. In IL-1β-induced articular cartilage inflammation, excessive activation of Wnt/β-catenin signaling has been reported, characterized by increased expression of Wnt1 and β-catenin together with reduced levels of its negative regulator GSK-3β. This dysregulation is closely associated with OA-like changes, including increased release of extracellular matrix degradation markers such as C-terminal cross-linked telopeptide of type II collagen (CTX-II) and cartilage oligomeric matrix protein, as well as marked disruption of chondrocyte ultrastructure (88). PRP treatment has been shown to reverse these abnormalities to an extent comparable to that achieved with the specific Wnt inhibitor Dickkopf-1 (Dkk-1), indicating that the chondroprotective effects of PRP are mediated not only by its growth factor content but also by its capacity to modulate pathological signaling (122). By attenuating IL-1β-induced overactivation of Wnt/β-catenin signaling, PRP may reduce chondrocyte apoptosis and matrix degradation (Table 7).

**Table 7:** Studies on PRP/PRP-derived exosomes and Wnt/β-catenin signaling in cartilage.

References	Model type (animal/human)	*In vivo*/*in vitro*	Study type	Type of cartilage injury	Model of injury (natural vs. induced)
Wu et al. (87)	Rabbit articular chondrocytes activated by IL-1β (animal)	*In vitro*	Basic research	Inflammatory cartilage injury induced by IL-1β	Induced (proinflammatory stimulation with IL-1β)
Liu et al. (88)	Rat knee OA model + chondrocytes treated with PRP-derived exosomes (animal)	*In vivo* (rat knee OA) + *in vitro* (chondrocytes)	Preclinical (animal) + basic research	Degenerative osteoarthritis-like articular cartilage damage	Induced OA (surgical induction)

To further elucidate the active components of PRP, recent studies have increasingly focused on PRP-Exos. As important mediators of intercellular communication, exosomes protect and deliver encapsulated cargo, including proteins and microRNAs, to target cells, thereby enabling more stable and targeted biological effects. Experimental evidence suggests that these extracellular vesicles may contain proteins, growth factors, and regulatory microRNAs capable of modulating chondrocyte proliferation, apoptosis, and inflammatory responses. For instance, several *in vitro* studies have reported that PRP-Exos can regulate components of the Wnt/β-catenin signaling pathway in IL-1β-induced inflammatory models of chondrocytes. However, these findings are derived predominantly from cell culture and animal studies. To date, clinical investigations evaluating PRP-Exos in OA or cartilage repair remain limited. Furthermore, the precise effector molecules contained within PRP-Exos and their direct molecular targets have not yet been fully characterized. In addition, the optimal dose, injection frequency, and long-term safety profile of PRP-Exos require systematic evaluation in animal models and, ultimately, in human clinical studies (121). Consequently, whether PRP-Exos confer additional therapeutic benefit beyond conventional PRP preparations in human patients remains uncertain and requires validation in well-designed clinical trials (89).

## 9 The role of the TGF-β1/Smad2/3 pathway in PRP

Multiple studies support a strong association between platelet concentration and the levels of growth factors in PRP, particularly PDGF and TGF-β1 (90, 91). *In vitro* studies have shown that inhibition of TGF-β1 signaling in nucleus pulposus cells reduces SMAD2/3 phosphorylation and suppresses matrix protein synthesis (122). By contrast, increasing TGF-β1 levels in platelet preparations has been shown to attenuate intervertebral disc degeneration in both *in vitro* and *in vivo* models, suggesting a protective effect on extracellular matrix homeostasis (92). These findings indicate that activation of the TGF-β1/SMAD2/3 pathway may be an important mechanism by which PRP delays or prevents disc degeneration and may also contribute to cartilage preservation.

**Figure 8:**
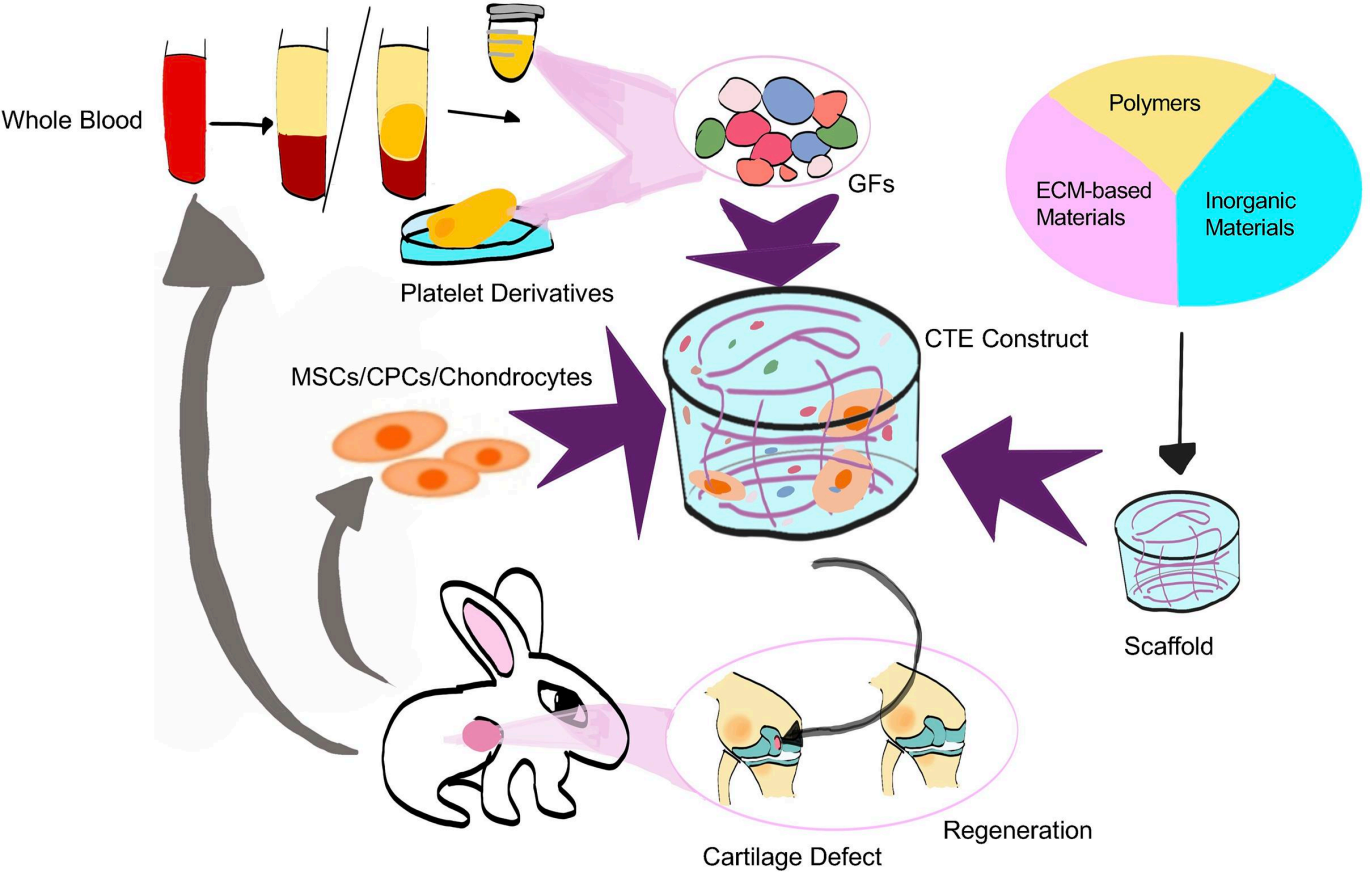
Composite of autologous platelet-rich plasma and a controlled-CTE scaffold for articular cartilage defect repair.

In the context of cartilage regeneration, TGF-β, one of the principal growth factors contained in PRP, has been shown to enhance ECM production in a manner comparable to that produced by dynamic mechanical loading. Pötter et al. reported that PRP, when used as an adjunct to autologous chondrocyte implantation, improved the expression of cartilage-specific markers and favorably modulated cytokine profiles under multiaxial loading conditions (Figure 8) (93). PRP also exerted anabolic effects on chondrocytes, including a marked increase in glycosaminoglycan synthesis, thereby highlighting its potential to support matrix assembly (123). Additional studies have demonstrated that the TGF-β/SMAD signaling pathway is a critical regulator of PRP-induced chondrogenesis in bone marrow–derived mesenchymal stem cells (BMSCs) (124). TGF-β released from PRP binds to TGF-β receptors on BMSCs, leading to SMAD2 phosphorylation and nuclear translocation, which, in turn, upregulates the transcription and translation of key chondrogenic markers, including SOX9, COL2A1, and ACAN (94).

Despite these promising mechanistic findings, further investigation is required to clarify how PRP-derived TGF-β signaling interacts with inflammatory pathways in joint tissues. In particular, the relative efficacy and safety of L-PRP and LP-PRP remain to be fully determined, as L-PRP may provide beneficial growth factor levels, while also carrying a greater burden of proinflammatory mediators (95). Future *in vitro* studies should systematically compare different PRP formulations, including their effects in coculture systems comprising not only articular chondrocytes but also synoviocytes and other intra-articular cell types, to better define the optimal preparations for clinical application (Table 8).

**Table 8:** Studies on the role of the TGF-β1/Smad2/3 pathway in PRP-related applications.

References	Model type (animal/human)	*In vivo*/*in vitro*	Study type	Type of cartilage injury	Model of injury (natural vs. induced)
Yang et al. (91)	Animal intervertebral disc degeneration model	*In vivo*	Preclinical (animal study)	Intervertebral disc degeneration	Induced degeneration
Pötter et al. (92)	MSC chondrogenesis model with PRP/platelet lysate and mechanical loading	*In vitro*	Basic research	Newly formed cartilage-like tissue *in vitro*	Induced (*in vitro* chondrogenic differentiation and mechanical loading)
Fang et al. (93)	MSC + collagen hydrogel + PRP, engineered cartilage implanted in animals	*In vitro* + *in vivo*	Basic + preclinical (animal implantation)	Traumatic focal cartilage defect repaired by engineered cartilage	Induced (surgically created cartilage defect followed by implantation)

## 10 Advances in clinical PRP therapies

Current evidence regarding the impact of PRP on outcomes after anterior cruciate ligament reconstruction (ACLR) remains heterogeneous and, at times, contradictory. In patients undergoing ACLR with hamstring tendon grafts, intraoperative application of PRP has been reported to accelerate graft maturation and improve anteroposterior knee stability at 6 months; however, it neither prevented femoral or tibial tunnel widening nor translated into superior postoperative functional scores (96). Several studies have also suggested that PRP may shorten the maturation period of patellar tendon–bone allografts, but without providing clear advantages in clinical function or biomechanical properties at 24-month follow-up (97, 98). Overall, the effect of PRP on tendon–bone integration after ACLR remains inconsistent, with reported outcomes ranging from no measurable benefit at the osteoligamentous interface to modest improvements in healing and vascularization at the graft–bone junction (99). Variability in PRP formulations, including LR-PRP vs. LP-PRP, together with differences in application techniques, dosing, and timing, likely contributes to these divergent findings and limits the strength of current clinical recommendations.

For medial collateral ligament (MCL) injuries, robust clinical evidence supporting intra-articular or periligamentous PRP application remains limited. Although some case series and small clinical studies have reported favorable outcomes with PRP in severe acute MCL lesions (100), a small comparative study found that PRP augmentation during arthroscopic MCL repair neither reduced reoperation rates nor hastened return to daily activities compared with standard repair alone (101). Larger, well-designed randomized controlled trials are required to establish whether PRP provides a clinically meaningful benefit in the treatment of MCL injuries.

The use of PRP in meniscal pathology has been investigated in a limited number of studies. A systematic review suggested that PRP injection may reduce pain, facilitate earlier return to sports, and limit lesion progression in intrasubstance (grade 2) meniscal tears over approximately 6 months of follow-up (102). In addition, one study reported that open meniscal repair augmented with PRP resulted in better outcomes than open repair alone, suggesting a potential role for PRP in enhancing meniscal healing in selected patients (103). Nevertheless, the heterogeneity of study designs, PRP formulations, and outcome measures precludes definitive conclusions.

Intra-articular PRP injections for KOA have generally demonstrated superior pain relief and functional improvement compared with saline and corticosteroid injections, particularly in younger patients with mild-to-moderate disease (104). Compared with hyaluronic acid (HA), PRP often provides greater symptomatic benefit, although results across different viscosupplementation regimens remain variable; one randomized controlled trial reported that the superiority of PRP over HA diminished after 9 months (105). Multiple PRP injections, commonly administered as two to three injections at intervals of 1–4 weeks, tend to yield better outcomes than viscosupplementation alone, whereas both single- and double-injection PRP protocols have been shown to be more effective than saline placebo (106). Adverse events are usually mild and transient, most commonly consisting of postinjection pain and swelling. Some studies suggest that LR-PRP is associated with a higher incidence of postinjection pain and synovitis than LP-PRP, without clear evidence of superior long-term benefit, thereby supporting the preferential use of leukocyte-poor preparations for intra-articular applications (107). In hip osteoarthritis, PRP appears to be safe and may provide short-term symptomatic relief, but it has not consistently demonstrated clear superiority over HA or other viscosupplementation treatments.

Despite its widespread clinical use, PRP therapy remains constrained by substantial variability in composition and preparation methods. The relationship between platelet count, fold increase in platelet concentration, and the actual levels and bioactivity of released growth factors is not linear and remains incompletely understood. Interindividual variation in baseline platelet counts, differences in the volume of blood collected, centrifugation protocols (e.g., single vs. double spin, including variations in speed and duration), activation methods, and the choice of anticoagulant (e.g., sodium citrate vs. ethylenediaminetetraacetic acid) all contribute to heterogeneity in the final PRP product (108). Furthermore, higher platelet concentrations do not necessarily translate into greater regenerative efficacy and may even be detrimental in certain contexts. Standardization of PRP preparation—including transparent reporting of platelet and leukocyte counts, activation status, and dosing regimens—is therefore essential to enable meaningful comparisons across studies and to optimize protocols for specific clinical indications. Future trials should systematically compare different PRP formulations, including leukocyte-rich and leukocyte-poor preparations, as well as different doses and injection schedules, to establish evidence-based recommendations for clinical practice.

Beyond symptomatic outcomes, several studies have sought to evaluate whether PRP therapy can affect structural changes in KOA as assessed by magnetic resonance imaging. Structural MRI parameters, including cartilage thickness, cartilage volume, bone marrow lesions, and synovial inflammation, are increasingly regarded as important indicators of disease progression.

Several clinical trials have examined these structural endpoints. For instance, the RESTORE randomized clinical trial evaluated intra-articular PRP injections compared with placebo in patients with symptomatic knee osteoarthritis and incorporated MRI-based assessment of medial tibial cartilage volume (125). Although PRP treatment was associated with improvements in pain and functional outcomes in some patients, the study did not demonstrate a significant between-group difference in the rate of cartilage volume loss over the follow-up period (126). These findings suggest that the symptomatic benefits of PRP may not necessarily correspond to measurable structural modification of cartilage degeneration on MRI within the time frame studied.

Other observational studies and smaller clinical trials have reported inconsistent findings. Some studies suggest that PRP may reduce synovial inflammation, improve cartilage signal intensity, or limit the progression of bone marrow lesions on MRI (127). However, these studies are frequently limited by small sample sizes, heterogeneous imaging protocols, and relatively short follow-up periods. Consequently, the current evidence remains insufficient to support the conclusion that PRP exerts a clear disease-modifying effect on joint structure in KOA.

Taken together, although PRP injections appear to provide symptomatic benefit in selected patients with mild-to-moderate KOA, current MRI-based studies have not consistently shown meaningful structural improvement in articular cartilage. Larger randomized controlled trials using standardized MRI assessment protocols and longer follow-up are required to determine whether PRP can genuinely alter the structural progression of OA.

## 11 Prospects for the future

The therapeutic potential of PRP may be further enhanced by combining it synergistically with complementary strategies. Emerging evidence suggests that the integration of PRP with dynamic mechanical loading, such as that generated by structured exercise or rehabilitation programs, and with biomaterials such as hyaluronic acid, may amplify its regenerative effects. The rationale underlying these combination approaches is to create a more favorable microenvironment for cartilage repair by coupling the biochemical cues delivered by PRP with beneficial mechanical stimulation and improved joint lubrication. Such multimodal strategies may represent a promising avenue for optimizing treatment protocols in osteoarthritis and other cartilage disorders; however, rigorous preclinical and clinical evaluation is required to define their efficacy, safety, and optimal implementation.

### 11.1 Combining PRP applications with exercise therapy

The role of mechanical loading in the context of PRP therapy for OA has attracted increasing research interest. Some evidence suggests that incorporating therapeutic joint loading, such as that achieved through structured exercise or rehabilitation programs, into PRP treatment protocols may improve clinical efficacy in OA (109). Exercise itself can transiently increase circulating platelet counts and alter systemic physiology, which may indirectly affect the biological activity of PRP (110). Nevertheless, the available evidence remains heterogeneous, and findings across studies are inconclusive.

Mechanical loading is a key regulator of chondrocyte behavior and plays a central role in both the maintenance of cartilage homeostasis and the pathogenesis of OA (111). Appropriate dynamic loading is considered essential for preserving articular cartilage function and may help slow, or even arrest, OA progression, whereas excessive or abnormal loading can accelerate degenerative changes.

In a retrospective cohort study, Kawahara et al. reported an additive, and potentially synergistic, effect of combining PRP injections with exercise therapy in patients with KOA (112). Over 12 months of follow-up, patients who received combined treatment with PRP and structured exercise experienced rapid pain and symptom relief comparable to that observed with PRP monotherapy at 1 month, while also demonstrating greater improvements in activities of daily living and quality of life from 3 to 12 months. The combined-treatment group achieved the highest OMERACT-OARSI responder rate at 12 months (65.0%), significantly higher than that of the PRP-only group (46.6%), suggesting a synergistic interaction between the two interventions. The authors proposed that the early anti-inflammatory and analgesic effects of PRP may facilitate patient engagement in exercise, which may subsequently promote longer-term functional gains by increasing muscle strength, improving proprioception, and enhancing movement patterns.

Based on these observations, it is plausible that the coordinated activation of mechanotransduction pathways through exercise, together with PRP-mediated biochemical modulation, may play a pivotal role in attenuating or even preventing OA progression. Nevertheless, further clinical studies are required to clarify how loading protocols within rehabilitation programs should be optimally designed and individualized to preserve joint health and maximize the therapeutic benefits of PRP in the context of joint inflammation.

### 11.2 Combining PRP application with HA

HA is an endogenous glycosaminoglycan found in synovial fluid, where it contributes to viscosity, elasticity, and joint lubrication. Intra-articular HA injections can partially restore these properties in damaged joints and have been shown to improve pain and function in KOA (113). Observational studies suggest that HA may delay the need for total knee arthroplasty by a median of up to 9.8 months, whereas PRP may potentially prolong this interval by approximately 4.1 years; however, these estimates are susceptible to confounding and should therefore be interpreted cautiously (114, 115). More recently, HA has been combined with PRP on the premise that their mechanisms of action may be complementary and capable of producing additive or synergistic effects (116). Several clinical studies suggest that PRP plus HA combination therapy in KOA may provide superior short- and long-term symptom control compared with either treatment alone, with some reports indicating that the full therapeutic effect may become apparent only after 52 weeks of follow-up (117).

Intra-articular administration of HA exerts both biomechanical and biological effects. From a biomechanical perspective, HA improves joint lubrication and shock absorption. Biologically, it can modulate inflammatory and nociceptive processes by reducing phagocytic activity, inhibiting proinflammatory mediators, and attenuating pain signaling (118). PRP, through growth factors contained within platelet α-granules, contributes to tissue repair not only by modulating inflammation but also by promoting angiogenesis, recruiting fibroblasts and mesenchymal stem cells to the lesion site, and stimulating autocrine growth factor production by resident cells (118). Accordingly, the combination of HA and PRP may provide both enhanced joint biomechanics and a more favorable biochemical environment for cartilage repair.

Nonetheless, high-quality randomized controlled trials employing standardized PRP and HA protocols and longer follow-up periods are required to more clearly define the long-term efficacy and safety of PRP plus HA combination therapy in KOA and other joint disorders, as well as to identify the patient subgroups most likely to benefit from this approach (128).

## 12 Discussion

PRP should be regarded not as a pathway-specific molecular therapy but as a heterogeneous biologic product that may modulate multiple signaling networks involved in cartilage degeneration and repair. The most consistent mechanistic concept emerging from preclinical studies is that PRP may shift the joint microenvironment away from a catabolic and inflammatory state toward one that is more permissive for repair. This includes attenuation of excessive NF-κB-driven inflammatory signaling, possible normalization of stress-associated Wnt/β-catenin and PI3K/AKT/mTOR activity, and promotion of anabolic responses associated with TGF-β/Smad signaling.

However, these mechanistic effects should not be conflated with the broader biology of signaling pathways involved in OA. A substantial proportion of the literature describes how Wnt, NF-κB, PI3K/AKT/mTOR, and TGF-β/Smad signaling function in osteoarthritis, whereas only a relatively limited number of studies directly demonstrate that PRP specifically modulates these pathways. Moreover, most mechanistic evidence continues to derive from *in vitro* and animal models, and the translational relationship between pathway regulation and clinical improvement in human patients remains insufficiently defined.

The biological efficacy of PRP is also highly dependent on its formulation. Platelet concentration, leukocyte content, red blood cell contamination, activation method, and dosing schedule may all influence whether the net effect is predominantly anti-inflammatory, anabolic, or, in certain settings, counterproductive. Standardized and transparent reporting of PRP composition and preparation is therefore essential for enabling meaningful comparisons across studies and for identifying the product characteristics most relevant to specific clinical indications.

From a clinical perspective, intra-articular PRP appears to provide symptomatic benefit in selected patients with mild-to-moderate KOA, particularly with respect to pain relief and functional improvement. By contrast, evidence for PRP in ligament and meniscal repair remains inconsistent, and current MRI-based studies have not conclusively demonstrated a reproducible structural disease-modifying effect on articular cartilage. Emerging approaches, such as PRP combined with exercise therapy, PRP plus HA, and PRP-Exos, remain promising but should still be considered investigational.

Several limitations of the current evidence base warrant acknowledgment. First, a substantial portion of the mechanistic literature is derived from preclinical studies and cannot be assumed to directly predict clinical outcomes. Second, many clinical studies are constrained by small sample sizes, heterogeneous patient populations, short follow-up durations, and non-standardized PRP protocols. Third, many published discussions of signaling pathways fail to clearly distinguish PRP-specific biological effects from the broader pathophysiology of OA, which may result in mechanistic overinterpretation. Finally, as a narrative review, the present manuscript does not provide a formal assessment of risk of bias and should therefore be interpreted with appropriate caution.

Future research should move beyond broad pathway descriptions and instead determine which PRP formulations, doses, delivery strategies, and patient phenotypes are associated with reproducible biological and clinical effects. Well-designed randomized controlled trials incorporating standardized PRP characterization, extended follow-up, and structural outcome measures will be essential to establish whether PRP can progress from a promising empirical intervention to a more mechanism-informed and evidence-based therapy for cartilage repair and the management of OA.

## Glossary

ECM: extracellular matrix
OA: osteoarthritis
MMPs: matrix metalloproteinases
PRP: platelet-rich plasma
IL-1β: interleukin-1β
TNF-α: tumor necrosis factor-α
NF-κB: nuclear factor-κB
PI3K/AKT/mTOR: phosphoinositide 3-kinase/protein kinase B/mammalian target of rapamycin
TGF-β: transforming growth factor-β
SFRP3: secreted frizzled-related protein 3
TCF/LEF: T-cell factor/lymphoid enhancer factor
GSK: glycogen synthase kinase
LiCl: lithium chloride
PCP: planar cell polarity
PRG: proteoglycan
PTHrP: parathyroid hormone-related protein
mTORC1: mammalian target of rapamycin complex
LPS: lipopolysaccharide
TLRs: toll-like receptors
IKK: IκB kinase
RANK: receptor activator of NF-κB
BAFF-R: B-cell-activating factor receptor
NIK: NF-κB-inducing kinase
SNPs: single-nucleotide polymorphisms
nNOS: neuronal nitric oxide synthase
TNFRs: tumor necrosis factor receptors
BMP: bone morphogenetic protein
ADAMTS: A disintegrin and metalloproteinase with thrombospondin motifs
PGE2: prostaglandin E2
COX-2: cyclooxygenase-2
GPCRs: G protein-coupled receptors
PI3K: phosphoinositide 3-kinase
PIP2: phosphatidylinositol 4,5-bisphosphate
PIP3: phosphatidylinositol 3,4,5-trisphosphate
PTEN: phosphatase and tensin homolog
PDK1: phosphoinositide-dependent protein kinase 1
S6K1: S6 kinase beta-1
4E-BP1: 4E-binding protein 1
ULK1: Unc-51-like kinase 1
TSC1/TSC2: tuberous sclerosis complex
Rheb: Ras homolog enriched in brain
IGF-1: insulin-like growth factor-1
Col2a1: type II collagen
ERK: extracellular signal-regulated kinase
GDFs: growth differentiation factors
R-SMADs: receptor-regulated SMADs
TAK1: transforming growth factor-β-activated kinase 1
ERK1/2: extracellular signal-regulated kinase 1/2
miRNAs: microRNAs
AMPK: AMP-activated protein kinase
MAPK: p38 mitogen-activated protein kinase
PDGF: platelet-derived growth factor
VEGF: vascular endothelial growth factor
EGF: epidermal growth factor
ACTH: adrenocorticotropic hormone
hGH: human growth hormone
MPS: mononuclear phagocyte system
FGF: fibroblast growth factor
RBCs: red blood cells
ROS: reactive oxygen species
LP-PRP: leukocyte-poor PRP
LR-PRP: leukocyte-rich PRP
HGF: hepatocyte growth factor
MCP-1: monocyte chemoattractant protein-1
PRP-Exos: PRP-derived exosomes
CTX-II: cross-linked telopeptide of type II collagen
COMP: cartilage oligomeric matrix protein
Dkk-1: Dickkopf-1
ACI: autologous chondrocyte implantation
BMSCs: bone marrow–derived mesenchymal stem cells
ACLR: anterior cruciate ligament reconstruction
MCL: medial collateral ligament
HA: hyaluronic acid
MRI: magnetic resonance imaging
ADL: activities of daily living
QOL: quality of life.

## References

[1] Wu S Guo W Li R Zhang X Qu W. Progress of platelet derivatives for cartilage tissue engineering. Front Bioeng Biotechnol. (2022) 10:907356. 10.3389/fbioe.2022.907356 35782516 PMC9243565

[2] Mumme M Wixmerten A Ivkovic A Peretti GM Yilmaz T Reppenhagen S Clinical relevance of engineered cartilage maturation in a randomized multicenter trial for articular cartilage repair. Sci Transl Med. (2025) 17(788):eads0848. 10.1126/scitranslmed.ads0848 40043142

[3] Kim KI Lee MC Lee JH Moon YW Lee WS Lee HJ Clinical efficacy and safety of the intra-articular injection of autologous adipose-derived mesenchymal stem cells for knee osteoarthritis: a phase III, randomized, double-blind, placebo-controlled trial. Am J Sports Med. (2023) 51(9):2243–53. 10.1177/03635465231179223 37345256

[4] Huang L Zhang S Wu J Guo B Gao T Shah SZA Immunity-and-matrix-regulatory cells enhance cartilage regeneration for meniscus injuries: a phase I dose-escalation trial. Signal Transduct Target Ther. (2023) 8(1):417. 10.1038/s41392-023-01670-7 37907503 PMC10618459

[5] Sadri B Hassanzadeh M Bagherifard A Mohammadi J Alikhani M Moeinabadi-Bidgoli K Cartilage regeneration and inflammation modulation in knee osteoarthritis following injection of allogeneic adipose-derived mesenchymal stromal cells: a phase II, triple-blinded, placebo controlled, randomized trial. Stem Cell Res Ther. (2023) 14(1):162. 10.1186/s13287-023-03359-8 37316949 PMC10268462

[6] Qiao J Guo X Zhang L Zhao H He X. Autologous platelet rich plasma injection can be effective in the management of osteoarthritis of the knee: impact on IL-1 *β*, TNF-α, hs-CRP. J Orthop Surg Res. (2024) 19(1):703. 10.1186/s13018-024-05060-9 39478604 PMC11523966

[7] Yoshioka T Arai N Sugaya H Taniguchi Y Kanamori A Gosho M The effectiveness of leukocyte-poor platelet-rich plasma injections for symptomatic mild to moderate osteoarthritis of the knee with joint effusion or bone marrow lesions in a Japanese population: a randomized, double-blind, placebo-controlled clinical trial. Am J Sports Med. (2024) 52(10):2493–502. 10.1177/03635465241263073 39097760

[8] Xiao Z Chen W Wei Z Zhang Q Tang G. Global trends and hotspots in the application of platelet-rich plasma in knee osteoarthritis: a bibliometric analysis from 2008 to 2022. Medicine. (2023) 102(47):e35854. 10.1097/MD.0000000000035854 38013292 PMC10681507

[9] Sun JY Li C Du FY. Adenylate cyclase activates the cAMP signalling pathway to enhance platelet-rich plasma-treated achilles tendon disease, a theoretical bioinformatics-based study. World J Orthop. (2024) 15(2):192–200. 10.5312/wjo.v15.i2.192 38464349 PMC10921184

[10] Du G Sun X He S Mi L. The Nrf2/HO-1 pathway participates in the antiapoptotic and anti-inflammatory effects of platelet-rich plasma in the treatment of osteoarthritis. Immun Inflamm Dis. (2024) 12(6):e1169. 10.1002/iid3.1169 38860757 PMC11165680

[11] Reya T Clevers H. Wnt signalling in stem cells and cancer. Nature. (2005) 434(7035):843–50. 10.1038/nature03319 15829953

[12] Schunk SJ Floege J Fliser D Speer T. WNT-*β*-catenin signalling—a versatile player in kidney injury and repair. Nat Rev Nephrol. (2021) 17(3):172–84. 10.1038/s41581-020-00343-w 32989282

[13] Nusse R Clevers H. Wnt/*β*-catenin signaling, disease, and emerging therapeutic modalities. Cell. (2017) 169(6):985–99. 10.1016/j.cell.2017.05.016 28575679

[14] Tong W Zeng Y Chow DHK Yeung W Xu J Deng Y Wnt16 attenuates osteoarthritis progression through a PCP/JNK-mTORC1-PTHrP cascade. Ann Rheum Dis. (2019) 78(4):551–61. 10.1136/annrheumdis-2018-214200 30745310

[15] Conde J Ruiz-Fernandez C Francisco V Scotece M Gómez R Lago F Dickkopf-3 (DKK3) signaling in IL-1*α*-challenged chondrocytes: involvement of the NF-*κ*B pathway. Cartilage. (2021) 13(2_suppl):925s–34. 10.1177/1947603520933328 32532182 PMC8804835

[16] Yasuhara R Ohta Y Yuasa T Kondo N Hoang T Addya S Roles of *β*-catenin signaling in phenotypic expression and proliferation of articular cartilage superficial zone cells. Lab Invest. (2011) 91(12):1739–52. 10.1038/labinvest.2011.144 21968810 PMC3759358

[17] Shepard JB Jeong JW Maihle NJ O'Brien S Dealy CN. Transient anabolic effects accompany epidermal growth factor receptor signal activation in articular cartilage *in vivo*. Arthritis Res Ther. (2013) 15(3):R60. 10.1186/ar4233 23705804 PMC4060279

[18] De Palma A Nalesso G. WNT signalling in osteoarthritis and its pharmacological targeting. Handb Exp Pharmacol. (2021) 269:337–56. 10.1007/164_2021_525 34510305

[19] Xia H Cao D Yang F Yang W Li W Liu P Jiawei Yanghe decoction ameliorates cartilage degradation *in vitro* and vivo via Wnt/*β*-catenin signaling pathway. Biomed Pharmacother. (2020) 122:109708. 10.1016/j.biopha.2019.109708 31918279

[20] Zhou Y Wang T Hamilton JL Chen D. Wnt/*β*-catenin signaling in osteoarthritis and in other forms of arthritis. Curr Rheumatol Rep. (2017) 19(9):53. 10.1007/s11926-017-0679-z 28752488 PMC5672801

[21] Snelling SJ Davidson RK Swingler TE Le LT Barter MJ Culley KL Dickkopf-3 is upregulated in osteoarthritis and has a chondroprotective role. Osteoarthritis Cartilage. (2016) 24(5):883–91. 10.1016/j.joca.2015.11.021 26687825 PMC4863878

[22] Hua B Qiu J Ye X Liu X. Intra-articular injection of a novel Wnt pathway inhibitor, SM04690, upregulates Wnt16 expression and reduces disease progression in temporomandibular joint osteoarthritis. Bone. (2022) 158:116372. 10.1016/j.bone.2022.116372 35218985

[23] Jimi E Ghosh S. Role of nuclear factor-kappaB in the immune system and bone. Immunol Rev. (2005) 208(1):80–7. 10.1111/j.0105-2896.2005.00329.x 16313342

[24] Yoon DS Lee KM Choi Y Ko EA Lee NH Cho S TLR4 downregulation by the RNA-binding protein PUM1 alleviates cellular aging and osteoarthritis. Cell Death Differ. (2022) 29(7):1364–78. 10.1038/s41418-021-00925-6 35034101 PMC9287402

[25] Kobayashi H Chang SH Mori D Itoh S Hirata M Hosaka Y Biphasic regulation of chondrocytes by Rela through induction of anti-apoptotic and catabolic target genes. Nat Commun. (2016) 7(1):13336. 10.1038/ncomms13336 27830706 PMC5109547

[26] Rahman MM Watton PN Neu CP Pierce DM. A chemo-mechano-biological modeling framework for cartilage evolving in health, disease, injury, and treatment. Comput Methods Programs Biomed. (2023) 231:107419. 10.1016/j.cmpb.2023.107419 36842346

[27] Ali SA Al-Jazrawe M Ma H Whetstone H Poon R Farr S Regulation of cholesterol homeostasis by hedgehog signaling in osteoarthritic cartilage. Arthritis Rheumatol. (2016) 68(1):127–37. 10.1002/art.39337 26315393 PMC4690757

[28] Liu X Jiang S Jiang T Lan Z Zhang X Zhong Z Bioenergetic-active exosomes for cartilage regeneration and homeostasis maintenance. Sci Adv. (2024) 10(42):eadp7872. 10.1126/sciadv.adp7872 39423269 PMC11488572

[29] Deng Y Lu J Li W Wu A Zhang X Tong W Reciprocal inhibition of YAP/TAZ and NF-*κ*B regulates osteoarthritic cartilage degradation. Nat Commun. (2018) 9(1):4564. 10.1038/s41467-018-07022-2 30385786 PMC6212432

[30] Chen S Qin L Wu X Fu X Lin S Chen D Moderate fluid shear stress regulates heme oxygenase-1 expression to promote autophagy and ECM homeostasis in the nucleus Pulposus cells. Front Cell Dev Biol. (2020) 8:127. 10.3389/fcell.2020.00127 32195253 PMC7064043

[31] Guo H German P Bai S Barnes S Guo W Qi X The PI3K/AKT pathway and renal cell carcinoma. J Genet Genomics. (2015) 42(7):343–53. 10.1016/j.jgg.2015.03.003 26233890 PMC4624215

[32] Vanhaesebroeck B Guillermet-Guibert J Graupera M Bilanges B. The emerging mechanisms of isoform-specific PI3K signalling. Nat Rev Mol Cell Biol. (2010) 11(5):329–41. 10.1038/nrm2882 20379207

[33] Huang J Manning BD. A complex interplay between Akt, TSC2 and the two mTOR complexes. Biochem Soc Trans. (2009) 37(1):217–22. 10.1042/BST0370217 19143635 PMC2778026

[34] Laplante M Sabatini DM. mTOR signaling in growth control and disease. Cell. (2012) 149(2):274–93. 10.1016/j.cell.2012.03.017 22500797 PMC3331679

[35] Dibble CC Cantley LC. Regulation of mTORC1 by PI3K signaling. Trends Cell Biol. (2015) 25(9):545–55. 10.1016/j.tcb.2015.06.002 26159692 PMC4734635

[36] Cravero JD Carlson CS Im HJ Yammani RR Long D Loeser RF. Increased expression of the Akt/PKB inhibitor TRB3 in osteoarthritic chondrocytes inhibits insulin-like growth factor 1-mediated cell survival and proteoglycan synthesis. Arthritis Rheum. (2009) 60(2):492–500. 10.1002/art.24225 19180501 PMC2637941

[37] Yao X Zhang J Jing X Ye Y Guo J Sun K Fibroblast growth factor 18 exerts anti-osteoarthritic effects through PI3K-AKT signaling and mitochondrial fusion and fission. Pharmacol Res. (2019) 139:314–24. 10.1016/j.phrs.2018.09.026 30273654

[38] Iwasa K Hayashi S Fujishiro T Kanzaki N Hashimoto S Sakata S PTEN Regulates matrix synthesis in adult human chondrocytes under oxidative stress. J Orthop Res. (2014) 32(2):231–7. 10.1002/jor.22506 24155249

[39] Rosa SC Rufino AT Judas F Tenreiro C Lopes MC Mendes AF. Expression and function of the insulin receptor in normal and osteoarthritic human chondrocytes: modulation of anabolic gene expression, glucose transport and GLUT-1 content by insulin. Osteoarthritis Cartilage. (2011) 19(6):719–27. 10.1016/j.joca.2011.02.004 21324373

[40] Venkatesan JK Rey-Rico A Schmitt G Wezel A Madry H Cucchiarini M. rAAV-mediated overexpression of TGF-*β* stably restructures human osteoarthritic articular cartilage in situ. J Transl Med. (2013) 11(1):211. 10.1186/1479-5876-11-211 24034904 PMC3847562

[41] Zhang M Zhou Q Liang QQ Li CG Holz JD Tang D IGF-1 regulation of type II collagen and MMP-13 expression in rat endplate chondrocytes via distinct signaling pathways. Osteoarthritis Cartilage. (2009) 17(1):100–6. 10.1016/j.joca.2008.05.007 18595745

[42] Hui W Litherland GJ Elias MS Kitson GI Cawston TE Rowan AD Leptin produced by joint white adipose tissue induces cartilage degradation via upregulation and activation of matrix metalloproteinases. Ann Rheum Dis. (2012) 71(3):455–62. 10.1136/annrheumdis-2011-200372 22072016

[43] Sun K Luo J Guo J Yao X Jing X Guo F. The PI3K/AKT/mTOR signaling pathway in osteoarthritis: a narrative review. Osteoarthritis Cartilage. (2020) 28(4):400–9. 10.1016/j.joca.2020.02.027 32081707

[44] Salazar VS Gamer LW Rosen V. BMP signalling in skeletal development, disease and repair. Nat Rev Endocrinol. (2016) 12(4):203–21. 10.1038/nrendo.2016.12 26893264

[45] Zhang Y Que J. BMP signaling in development, stem cells, and diseases of the gastrointestinal tract. Annu Rev Physiol. (2020) 82(1):251–73. 10.1146/annurev-physiol-021119-034500 31618602 PMC13090088

[46] Wang RN Green J Wang Z Deng Y Qiao M Peabody M Bone morphogenetic protein (BMP) signaling in development and human diseases. Genes Dis. (2014) 1(1):87–105. 10.1016/j.gendis.2014.07.005 25401122 PMC4232216

[47] Lowery JW Rosen V. The BMP pathway and its inhibitors in the skeleton. Physiol Rev. (2018) 98(4):2431–52. 10.1152/physrev.00028.2017 30156494

[48] Thielen NGM van der Kraan PM van Caam APM. TGF*β*/BMP signaling pathway in cartilage homeostasis. Cells. (2019) 8(9):969. 10.3390/cells8090969 31450621 PMC6769927

[49] Hiepen C Mendez PL Knaus P. It takes two to Tango: endothelial TGF*β*/BMP signaling crosstalk with mechanobiology. Cells. (2020) 9:1965. 10.3390/cells9091965 32858894 PMC7564048

[50] Luo Z Jiang L Xu Y Li H Xu W Wu S Mechano growth factor (MGF) and transforming growth factor (TGF)-β3 functionalized silk scaffolds enhance articular hyaline cartilage regeneration in rabbit model. Biomaterials. (2015) 52:463–75. 10.1016/j.biomaterials.2015.01.001 25818452

[51] Lu W He Z Shi J Wang Z Wu W Liu J AMD3100 attenuates post-traumatic osteoarthritis by maintaining transforming growth factor-*β*1-induced expression of tissue inhibitor of metalloproteinase-3 via the phosphatidylinositol 3-kinase/akt pathway. Front Pharmacol. (2019) 10:1554. 10.3389/fphar.2019.01554 32038242 PMC6987846

[52] Chavez RD Coricor G Perez J Seo HS Serra R. SOX9 Protein is stabilized by TGF-*β* and regulates PAPSS2 mRNA expression in chondrocytes. Osteoarthritis Cartilage. (2017) 25:332–40. 10.1016/j.joca.2016.10.007 27746378 PMC5258840

[53] Boyan BD Hyzy SL Pan Q Scott KM Coutts RD Healey R 24R,25-dihydroxyvitamin D3 protects against articular cartilage damage following anterior cruciate ligament transection in male rats. PLoS One. (2016) 11(8):e0161782. 10.1371/journal.pone.0161782 27575371 PMC5019362

[54] Chavez RD Sohn P Serra R. Prg4 prevents osteoarthritis induced by dominant-negative interference of TGF-ß signaling in mice. PLoS One. (2019) 14(1):e0210601. 10.1371/journal.pone.0210601 30629676 PMC6328116

[55] Yang X Guan Y Tian S Wang Y Sun K Chen Q. Mechanical and IL-1β responsive miR-365 contributes to osteoarthritis development by targeting histone deacetylase 4. Int J Mol Sci. (2016) 17(4):436. 10.3390/ijms17040436 27023516 PMC4848892

[56] Yao Q Wu X Tao C Gong W Chen M Qu M Osteoarthritis: pathogenic signaling pathways and therapeutic targets. Signal Transduct Target Ther. (2023) 8(1):56. 10.1038/s41392-023-01330-w 36737426 PMC9898571

[57] Ghoshal K Bhattacharyya M. Overview of platelet physiology: its hemostatic and nonhemostatic role in disease pathogenesis. ScientificWorldJournal. (2014) 2014:781857. 10.1155/2014/781857 24729754 PMC3960550

[58] Derwich M Mitus-Kenig M Pawlowska E. Mechanisms of action and efficacy of hyaluronic acid, corticosteroids and platelet-rich plasma in the treatment of temporomandibular joint osteoarthritis-A systematic review. Int J Mol Sci. (2021) 22(14):7405. 10.3390/ijms22147405 34299024 PMC8308010

[59] Raum G Kenyon C Bowers R. Platelet-poor versus platelet-rich plasma for the treatment of muscle injuries. Curr Sports Med Rep. (2024) 23:222–8. 10.1249/JSR.0000000000001173 38838685

[60] Markopoulou CE Markopoulos P Dereka XE Pepelassi E Vrotsos IA. Effect of homologous PRP on proliferation of human periodontally affected osteoblasts. *In vitro* preliminary study. Report of a case. J Musculoskelet Neuronal Interact. (2009) 9:167–72. 19724151

[61] Yamaguchi R Terashima H Yoneyama S Tadano S Ohkohchi N. Effects of platelet-rich plasma on intestinal anastomotic healing in rats: PRP concentration is a key factor. J Surg Res. (2012) 173:258–66. 10.1016/j.jss.2010.10.001 21074782

[62] Rui S Yuan Y Du C Song P Chen Y Wang H Comparison and investigation of exosomes derived from platelet-rich plasma activated by different agonists. Cell Transplant. (2021) 30:9636897211017833. 10.1177/09636897211017833 34006140 PMC8138303

[63] Saumell-Esnaola M Delgado D García Del Caño G Beitia M Sallés J González-Burguera I Isolation of platelet-derived exosomes from human platelet-rich plasma: biochemical and morphological characterization. Int J Mol Sci. (2022) 23:2861. 10.3390/ijms23052861 35270001 PMC8911307

[64] Anderson NL Anderson NG. The human plasma proteome: history, character, and diagnostic prospects. Mol Cell Proteomics. (2002) 1(11):845–67. 10.1074/mcp.R200007-MCP200 12488461

[65] Deutsch EW Omenn GS Sun Z Maes M Pernemalm M Palaniappan KK Advances and utility of the human plasma proteome. J Proteome Res. (2021) 20(12):5241–63. 10.1021/acs.jproteome.1c00657 34672606 PMC9469506

[66] Everts P Onishi K Jayaram P Lana JF Mautner K. Platelet-rich plasma: new performance understandings and therapeutic considerations in 2020. Int J Mol Sci. (2020) 21:7794. 10.3390/ijms21207794 33096812 PMC7589810

[67] Kang J Yang L Jia T Zhang W Wang LB Zhao YJ Plasma proteomics identifies proteins and pathways associated with incident depression in 46,165 adults. Sci Bull. (2025) 70:573–86. 10.1016/j.scib.2024.09.041 39424455

[68] Fuentes-Lemus E Reyes JS López-Alarcón C Davies MJ. Crowding modulates the glycation of plasma proteins: *in vitro* analysis of structural modifications to albumin and transferrin and identification of sites of modification. Free Radic Biol Med. (2022) 193:551–66. 10.1016/j.freeradbiomed.2022.10.319 36336230

[69] Ragni E. Extracellular vesicles: recent advances and perspectives. Front Biosci. (2025) 30:36405. 10.31083/FBL36405 40613286

[70] Hoemann CD Chen G Marchand C Tran-Khanh N Thibault M Chevrier A Scaffold-guided subchondral bone repair: implication of neutrophils and alternatively activated arginase-1+ macrophages. Am J Sports Med. (2010) 38:1845–56. 10.1177/0363546510369547 20522834

[71] Phillipson M Kubes P. The healing power of neutrophils. Trends Immunol. (2019) 40:635–47. 10.1016/j.it.2019.05.001 31160208

[72] Adrover JM Aroca-Crevillén A Crainiciuc G Ostos F Rojas-Vega Y Rubio-Ponce A Programmed ‘disarming’ of the neutrophil proteome reduces the magnitude of inflammation. Nat Immunol. (2020) 21:135–44. 10.1038/s41590-019-0571-2 31932813 PMC7223223

[73] Shimizu Y Ntege EH Sunami H Inoue Y. Regenerative medicine strategies for hair growth and regeneration: a narrative review of literature. Regen Ther. (2022) 21:527–39. 10.1016/j.reth.2022.10.005 36382136 PMC9637724

[74] Nishio H Saita Y Kobayashi Y Takaku T Fukusato S Uchino S Platelet-rich plasma promotes recruitment of macrophages in the process of tendon healing. Regen Ther. (2020) 14:262–70. 10.1016/j.reth.2020.03.009 32455156 PMC7232040

[75] Chen T Song P He M Rui S Duan X Ma Y Sphingosine-1-phosphate derived from PRP-exos promotes angiogenesis in diabetic wound healing via the S1PR1/AKT/FN1 signalling pathway. Burns Trauma. (2023) 11:tkad003. 10.1093/burnst/tkad003 37251708 PMC10208895

[76] Vladulescu D Scurtu LG Simionescu AA Scurtu F Popescu MI Simionescu O. Platelet-rich plasma (PRP) in dermatology: cellular and molecular mechanisms of action. Biomedicines. (2023) 12(1):7. 10.3390/biomedicines12010007 38275368 PMC10813350

[77] Amable PR Carias RB Teixeira MV da Cruz Pacheco I Corrêa do Amaral RJ Granjeiro JM Platelet-rich plasma preparation for regenerative medicine: optimization and quantification of cytokines and growth factors. Stem Cell Res Ther. (2013) 4:67. 10.1186/scrt218 23759113 PMC3706762

[78] Lee TY Lu WJ Changou CA Hsiung YC Trang NTT Lee CY Platelet autophagic machinery involved in thrombosis through a novel linkage of AMPK-MTOR to sphingolipid metabolism. Autophagy. (2021) 17:4141–58. 10.1080/15548627.2021.1904495 33749503 PMC8726689

[79] Saqlain N Mazher N Fateen T Siddique A. Comparison of single and double centrifugation methods for preparation of platelet-rich plasma (PRP). Pak J Med Sci. (2023) 39:634–7. 10.12669/pjms.39.3.7264 37250535 PMC10214802

[80] Harrison TE Bowler J Levins TN Reeves KD Cheng AL. Platelet-rich plasma centrifugation changes leukocyte ratios. Cureus. (2021) 13:e14470. 10.7759/cureus.14470 33996329 PMC8115186

[81] Cheng L Chang S Tan Y He B. Platelet-rich plasma combined with isometric quadriceps contraction regulates autophagy in chondrocytes via the PI3K/AKT/mTOR pathway to promote cartilage repair in knee osteoarthritis. Regen Ther. (2025) 28:81–9. 10.1016/j.reth.2024.11.013 39703816 PMC11655694

[82] Zhao H Zhu W Mao W Shen C. Platelet-rich plasma inhibits Adriamycin-induced inflammation via blocking the NF-*κ*B pathway in articular chondrocytes. Mol Med. (2021) 27:66. 10.1186/s10020-021-00314-2 34172007 PMC8229346

[83] Zhang L Zhang Q Cui L Wu L Gao S. Kartogenin combined platelet-rich plasma (PRP) promoted tendon-bone healing for anterior cruciate ligament (ACL) reconstruction by suppressing inflammatory response via targeting AKT/PI3K/NF-*κ*B. Appl Biochem Biotechnol. (2023) 195:1284–96. 10.1007/s12010-022-04178-y 36346560

[84] Bendinelli P Matteucci E Dogliotti G Corsi MM Banfi G Maroni P Molecular basis of anti-inflammatory action of platelet-rich plasma on human chondrocytes: mechanisms of NF-*κ*B inhibition via HGF. J Cell Physiol. (2010) 225:757–66. 10.1002/jcp.22274 20568106

[85] Xu J Chen X Zhang H Zhang X Liu R Li X Platelet-rich plasma relieves inflammation and pain by regulating M1/M2 macrophage polarization in knee osteoarthritis rats. Sci Rep. (2025) 15:12805. 10.1038/s41598-025-97501-6 40229323 PMC11997200

[86] Yin W Xu H Sheng J Xu Z Xie X Zhang C. Comparative evaluation of the effects of platelet-rich plasma formulations on extracellular matrix formation and the NF-*κ*B signaling pathway in human articular chondrocytes. Mol Med Rep. (2017) 15:2940–8. 10.3892/mmr.2017.6365 28339078 PMC5428536

[87] Wu J Huang JF Qin XX Hu F Chen ZF Zheng Y Platelet-rich plasma inhibits Wnt/*β*-catenin signaling in rabbit cartilage cells activated by IL-1β. Int Immunopharmacol. (2018) 55:282–9. 10.1016/j.intimp.2017.12.031 29291543

[88] Liu X Wang L Ma C Wang G Zhang Y Sun S. Exosomes derived from platelet-rich plasma present a novel potential in alleviating knee osteoarthritis by promoting proliferation and inhibiting apoptosis of chondrocyte via Wnt/*β*-catenin signaling pathway. J Orthop Surg Res. (2019) 14:470. 10.1186/s13018-019-1529-7 31888697 PMC6936129

[89] Mochizuki T Ushiki T Watanabe S Omori G Kawase T. The levels of TGF*β*1, VEGF, PDGF-BB, and PF4 in platelet-rich plasma of professional soccer players: a cross-sectional pilot study. J Orthop Surg Res. (2022) 17:465. 10.1186/s13018-022-03362-4 36303196 PMC9615199

[90] Solakoglu Ö Heydecke G Amiri N Anitua E. The use of plasma rich in growth factors (PRGF) in guided tissue regeneration and guided bone regeneration. A review of histological, immunohistochemical, histomorphometrical, radiological and clinical results in humans. Ann Anat. (2020) 231:151528. 10.1016/j.aanat.2020.151528 32376297

[91] Yang H Yuan C Wu C Qian J Shi Q Li X The role of TGF-*β*1/Smad2/3 pathway in platelet-rich plasma in retarding intervertebral disc degeneration. J Cell Mol Med. (2016) 20(8):1542–9. 10.1111/jcmm.12847 27061332 PMC4956937

[92] Pötter N Westbrock F Grad S Alini M Stoddart MJ Schmal H Evaluation of the influence of platelet-rich plasma (PRP), platelet lysate (PL) and mechanical loading on chondrogenesis *in vitro*. Sci Rep. (2021) 11:20188. 10.1038/s41598-021-99614-0 34642434 PMC8510996

[93] Fang D Jin P Huang Q Yang Y Zhao J Zheng L. Platelet-rich plasma promotes the regeneration of cartilage engineered by mesenchymal stem cells and collagen hydrogel via the TGF-*β*/SMAD signaling pathway. J Cell Physiol. (2019) 234:15627–37. 10.1002/jcp.28211 30768719

[94] Titan A Schär M Hutchinson I Demange M Chen T Rodeo S. Growth factor delivery to a cartilage-cartilage interface using platelet-rich concentrates on a hyaluronic acid scaffold. Arthroscopy. (2020) 36:1431–40. 10.1016/j.arthro.2019.12.004 31862290

[95] Sánchez M Anitua E Azofra J Prado R Muruzabal F Andia I. Ligamentization of tendon grafts treated with an endogenous preparation rich in growth factors: gross morphology and histology. Arthroscopy. (2010) 26:470–80. 10.1016/j.arthro.2009.08.019 20362825

[96] Vogrin M Rupreht M Crnjac A Dinevski D Krajnc Z Recnik G. The effect of platelet-derived growth factors on knee stability after anterior cruciate ligament reconstruction: a prospective randomized clinical study. Wien Klin Wochenschr. (2010) 122(Suppl 2):91–5. 10.1007/s00508-010-1340-2 20517680

[97] Rodríguez-Merchán EC. Anterior cruciate ligament reconstruction: is biological augmentation beneficial? Int J Mol Sci. (2021) 22:12566. 10.3390/ijms222212566 34830448 PMC8625610

[98] Sinkler MA Furdock RJ McMellen CJ Calcei JG Voos JE. Biologics, stem cells, growth factors, platelet-rich plasma, hemarthrosis, and scaffolds may enhance anterior cruciate ligament surgical treatment. Arthroscopy. (2023) 39:166–75. 10.1016/j.arthro.2022.11.006 36370920

[99] Zou G Zheng M Chen W He X Cang D. Autologous platelet-rich plasma therapy for refractory pain after low-grade medial collateral ligament injury. J Int Med Res. (2020) 48:300060520903636. 10.1177/0300060520903636 32090668 PMC7111026

[100] Amar E Snir N Sher O Brosh T Khashan M Salai M Platelet-rich plasma did not improve early healing of medial collateral ligament in rats. Arch Orthop Trauma Surg. (2015) 135:1571–7. 10.1007/s00402-015-2306-7 26298561

[101] Elphingstone JW Alston ET Colorado BS. Platelet-rich plasma for nonoperative management of degenerative meniscal tears: a systematic review. J Orthop. (2024) 54:67–75. 10.1016/j.jor.2024.03.009 39036807 PMC11259654

[102] Alkhuzai A Arif Getta H Ibrahim Mohammed A Aziz RS. Evaluation of orthobiological ozonized platelet-rich plasma therapy post-arthroscopic suturing and lone partial meniscectomy in the treatment of meniscal tears within degenerative knee osteoarthritis. Knee. (2024) 50:69–76. 10.1016/j.knee.2024.07.016 39128172

[103] Bennell KL Paterson KL Metcalf BR Duong V Eyles J Kasza J Effect of intra-articular platelet-rich plasma vs placebo injection on pain and medial tibial cartilage volume in patients with knee osteoarthritis: the RESTORE randomized clinical trial. Jama. (2021) 326:2021–30. 10.1001/jama.2021.19415 34812863 PMC8611484

[104] Bansal H Leon J Pont JL Wilson DA Bansal A Agarwal D Platelet-rich plasma (PRP) in osteoarthritis (OA) knee: correct dose critical for long term clinical efficacy. Sci Rep. (2021) 11:3971. 10.1038/s41598-021-83025-2 33597586 PMC7889864

[105] Raeissadat SA Ghazi Hosseini P Bahrami MH Roghani RS Fathi M Ahangar AG The comparison effects of intra-articular injection of platelet rich plasma (PRP), plasma rich in growth factor (PRGF), hyaluronic acid (HA), and ozone in knee osteoarthritis; a one year randomized clinical trial. BMC Musculoskelet Disord. (2021) 22:134. 10.1186/s12891-021-04017-x 33536010 PMC7860007

[106] Nouri F Babaee M Peydayesh P Esmaily H Raeissadat SA. Comparison between the effects of ultrasound guided intra-articular injections of platelet-rich plasma (PRP), high molecular weight hyaluronic acid, and their combination in hip osteoarthritis: a randomized clinical trial. BMC Musculoskelet Disord. (2022) 23:856. 10.1186/s12891-022-05787-8 36096771 PMC9464606

[107] Gardashli M Baron M Huang C Kaplan LD Meng Z Kouroupis D Mechanical loading and orthobiologic therapies in the treatment of post-traumatic osteoarthritis (PTOA): a comprehensive review. Front Bioeng Biotechnol. (2024) 12:1401207. 10.3389/fbioe.2024.1401207 38978717 PMC11228341

[108] Gamez C Schneider-Wald B Schuette A Mack M Hauk L Khan AUM Bioreactor for mobilization of mesenchymal stem/stromal cells into scaffolds under mechanical stimulation: preliminary results. PLoS One. (2020) 15:e0227553. 10.1371/journal.pone.0227553 31923210 PMC6953860

[109] Wang H Sun W Zhao D. Platelet-rich plasma combined with exercise therapy: a treatment option for knee osteoarthritis. Asian J Surg. (2023) 46:1382–3. 10.1016/j.asjsur.2022.08.125 36117059

[110] Jayaram P Liu C Dawson B Ketkar S Patel SJ Lee BH Leukocyte-dependent effects of platelet-rich plasma on cartilage loss and thermal hyperalgesia in a mouse model of post-traumatic osteoarthritis. Osteoarthritis Cartilage. (2020) 28:1385–93. 10.1016/j.joca.2020.06.004 32629163 PMC7787501

[111] Kawahara T Iida S Isoda K Kim S. Effects of platelet-rich plasma combined with exercise therapy for one year on knee osteoarthritis: retrospective cohort study. J Orthop Surg Res. (2024) 19:696. 10.1186/s13018-024-05186-w 39465403 PMC11514950

[112] Shtroblia V Petakh P Kamyshna I Halabitska I Kamyshnyi O. Recent advances in the management of knee osteoarthritis: a narrative review. Front Med. (2025) 12:1523027. 10.3389/fmed.2025.1523027 PMC11790583 39906596

[113] Bliddal H Beier J Hartkopp A Conaghan PG Henriksen M. Polyacrylamide gel versus hyaluronic acid for the treatment of knee osteoarthritis: a randomised controlled study. Clin Exp Rheumatol. (2024) 42:1729–35. 10.55563/clinexprheumatol/i3fqee 38525999

[114] Rasmussen S Petersen KK Aboo C Andersen JS Skjoldemose E Jørgensen NK Intra-articular injection of gold micro-particles with hyaluronic acid for painful knee osteoarthritis. BMC Musculoskelet Disord. (2024) 25:211. 10.1186/s12891-024-07321-4 38475764 PMC10935980

[115] Dulic O Rasovic P Lalic I Kecojevic V Gavrilovic G Abazovic D Bone marrow aspirate concentrate versus platelet rich plasma or hyaluronic acid for the treatment of knee osteoarthritis. Medicina (B Aires). (2021) 57:1193. 10.3390/medicina57111193 PMC8623697 34833411

[116] Oon SF Lazarakis S Mallawa G Nguyen C. Intra-articular hyaluronic acid and platelet-rich plasma as monotherapy or combination therapy in knee osteoarthritis? Regen Med. (2024) 19:637–44. 10.1080/17460751.2024.2439221 39663604 PMC11702996

[117] Ghorbani O Mahdibarzi D Yousefi-Tooddeshki P. Comparison of the short-term effect of intra-articular hyaluronic acid and platelet-rich plasma injections in knee osteoarthritis: a randomized clinical trial. J Prev Med Hyg. (2024) 65:E214–20. 10.15167/2421-4248/jpmh2024.65.2.3270 39430992 PMC11487735

[118] Fossati C Randelli FMN Sciancalepore F Maglione D Pasqualotto S Ambrogi F Efficacy of intra-articular injection of combined platelet-rich-plasma (PRP) and hyaluronic acid (HA) in knee degenerative joint disease: a prospective, randomized, double-blind clinical trial. Arch Orthop Trauma Surg. (2024) 144:5039–51. 10.1007/s00402-024-05603-z 39367905

[119] Liu X Wang Y Wen X Hao C Ma J Yan L. Platelet rich plasma alleviates endometritis induced by lipopolysaccharide in mice via inhibiting TLR4/NF-*κ*B signaling pathway. Am J Reprod Immunol. (2024) 91:E13833. 10.1111/aji.13833 38467595

[120] Araya N Miyatake K Tsuji K Katagiri H Nakagawa Y Hoshino T Intra-articular injection of pure platelet-rich plasma is the most effective treatment for joint pain by modulating synovial inflammation and calcitonin gene-related peptide expression in a rat arthritis model. Am J Sports Med. (2020) 48:2004–12. 10.1177/0363546520924011 32519886

[121] Dong B Liu X Li J Wang B Yin J Zhang H Berberine encapsulated in exosomes derived from platelet-rich plasma promotes chondrogenic differentiation of the bone marrow mesenchymal stem cells via the Wnt/*β*-catenin pathway. Biol Pharm Bull. (2022) 45:1444–51. 10.1248/bpb.b22-00206 35858798

[122] Mietsch A Neidlinger-Wilke C Schrezenmeier H Mauer UM Friemert B Wilke HJ Evaluation of platelet-rich plasma and hydrostatic pressure regarding cell differentiation in nucleus pulposus tissue engineering. J Tissue Eng Regen Med. (2013) 7:244–52. 10.1002/term.524 22162329

[123] Cardoneanu A Macovei LA Burlui AM Mihai IR Bratoiu I Rezus II Temporomandibular joint osteoarthritis: pathogenic mechanisms involving the cartilage and subchondral bone, and potential therapeutic strategies for joint regeneration. Int J Mol Sci. (2022) 24:171. 10.3390/ijms24010171 36613615 PMC9820477

[124] Luo D Ran Z Wu J Wang L Wu W Xie K MYC-mediated osseous regeneration via BMSCs/PRP/*β*-TCP/PCL bioprinted constructs: rapid defect rehabilitation and preliminary clinical efficacy evaluation. IUBMB Life. (2025) 77:e70036. 10.1002/iub.70036 40718956

[125] Oeding JF Varady NH Fearington FW Pareek A Strickland SM Nwachukwu BU Platelet-rich plasma versus alternative injections for osteoarthritis of the knee: a systematic review and statistical fragility index-based meta-analysis of randomized controlled trials. Am J Sports Med. (2024) 52(12):3147–60. 10.1177/03635465231224463 38420745

[126] Cobianchi Bellisari F De Marino L Arrigoni F Mariani S Bruno F Palumbo P T2-mapping MRI evaluation of patellofemoral cartilage in patients submitted to intra-articular platelet-rich plasma (PRP) injections. Radiol Med. (2021) 126:1085–94. 10.1007/s11547-021-01372-6 34008045 PMC8292236

[127] Sekiya I Katano H Mizuno M Endo K Asami A Kajiwara M 3D-MRI analysis of cartilage thickness changes after PRP injection in medial knee osteoarthritis: a preliminary report. PLoS One. (2025) 20(4):E0321067. 10.1371/journal.pone.0321067 40305563 PMC12043159

[128] Belk JW Kraeutler MJ Houck DA Goodrich JA Dragoo JL McCarty EC. Platelet-rich plasma versus hyaluronic acid for knee osteoarthritis: a systematic review and meta-analysis of randomized controlled trials. Am J Sports Med. (2021) 49(1):249–60. 10.1177/0363546520909397 32302218

[129] McLarnon M Heron N. Intra-articular platelet-rich plasma injections versus intra-articular corticosteroid injections for symptomatic management of knee osteoarthritis: systematic review and meta-analysis. BMC Musculoskelet Disord. (2021) 22(1):550. 10.1186/s12891-021-04308-3 34134679 PMC8208610

[130] Tatsuo T Okumo T Kachi I Iida Y Nishio T Okada H Preventive effect of platelet-rich plasma on fracture healing in a rat tibial nonunion model: a controlled laboratory experiment. Cureus. (2025) 17:e97823. 10.7759/cureus.97823 41458709 PMC12741677

[131] Devereaux J Nurgali K Kiatos D Sakkal S Apostolopoulos V. Effects of platelet-rich plasma and platelet-poor plasma on human dermal fibroblasts. Maturitas. (2018) 117:34–44. 10.1016/j.maturitas.2018.09.001 30314559

[132] Sidiropoulou S Papadaki S Tsouka AN Koutsaliaris IK Chantzichristos VG Pantazi D The effect of platelet-rich plasma on endothelial progenitor cell functionality. Angiology. (2021) 72(8):776–86. 10.1177/0003319721998895 33678047

[133] Dashore S Chouhan K Nanda S Sharma A. Preparation of platelet-rich plasma: national IADVL PRP taskforce recommendations. Indian Dermatol Online J. (2021) 12(7):S12–23. 10.4103/idoj.idoj_269_21 34976877 PMC8664176

[134] Schmauch E Severin Y Xing X Mangold A Conrad C Johansen P Targeting IL-1 controls refractory pityriasis rubra pilaris. Sci Adv. (2024) 10(27):eado2365. 10.1126/sciadv.ado2365 38959302 PMC11221491

[135] Pensato R Al-Amer R La Padula S. Protocol for obtaining platelet-rich plasma (PRP), platelet-poor plasma (PPP), and thrombin for autologous use. Aesthetic Plast Surg. (2024) 36:2594–5. 10.1007/s00266-023-03470-4 37438664

[136] Brown K Mendell J Ohwada S Hsu C He L Warren V Tolerability, pharmacokinetics, and pharmacodynamics of mirogabalin in healthy subjects: results from phase 1 studies. Pharmacol Res Perspect. (2018) 6(5):e00418. 10.1002/prp2.418 30151212 PMC6106189

[137] Collins T Alexander D Barkatali B. Platelet-rich plasma: a narrative review. EFORT Open Rev. (2021) 6(4):225–35. 10.1302/2058-5241.6.200017 34040800 PMC8142058

[138] Pablo-Torres C Delgado-Dolset MI Sanchez-Solares J Mera-Berriatua L Núñez Martín Buitrago L Reaño Martos M A method based on plateletpheresis to obtain functional platelet, CD3+ and CD14+ matched populations for research immunological studies. Clin Exp Allergy. (2022) 52(10):1157–68. 10.1111/cea.14192 35757844 PMC9796013

[139] Sanak F Iselin K Kaufmann C Bachmann LM Buhl D Parts J Optimized autologous Serum and activated leukocyte-poor platelet-rich plasma eye drops production protocol: effect of fasting, filtering, and complement inactivation on the concentration of growth factors and lipids. J Ocul Pharmacol Ther. (2026) 42(1):45–53. 10.1177/10807683251370853 40865098

[140] Yadav S. Discussing the debate: leukocyte-rich platelet-rich plasma versus leukocyte-poor platelet-rich plasma. Cureus. (2024) 16(4):e58381. 10.7759/cureus.58381 38756260 PMC11097227

[141] Di Martino A Boffa A Andriolo L Romandini I Altamura SA Cenacchi A Leukocyte-rich versus leukocyte-poor platelet-rich plasma for the treatment of knee osteoarthritis: a double-blind randomized trial. Am J Sports Med. (2022) 50(3):609–17. 10.1177/03635465211064303 35103547

[142] Jayaram P Mitchell PJT Shybut TB Moseley BJ Lee B. Leukocyte-rich platelet-rich plasma is predominantly anti-inflammatory compared with leukocyte-poor platelet-rich plasma in patients with mild-moderate knee osteoarthritis: a prospective, descriptive laboratory study. Am J Sports Med. (2023) 51(8):2133–40. 10.1177/03635465231170394 37199381

[143] Costa FR de Souza SAL Martins RA Costa BR Pires L de Macedo AP The role of injectable platelet-rich fibrin in orthopedics: where do we stand? Curr Issues Mol Biol. (2025) 47(4):239. 10.3390/cimb47040239 40699638 PMC12025550

[144] Whitney KE Dornan GJ King J Chahla J Evans TA Philippon MJ The effect of a single freeze-thaw cycle on matrix metalloproteinases in different human platelet-rich plasma formulations. Biomedicines. (2021) 9(10):1403. 10.3390/biomedicines9101403 34680520 PMC8533272

[145] Saita Y Kobayashi Y Uchino S Nishio H Wakayama T Fukusato S Platelet-rich plasma therapy for knee osteoarthritis: insights from real-world clinical data in Japan. Regen Ther. (2025) 29:427–34. 10.1016/j.reth.2025.04.002 40487921 PMC12144911

[146] Xu B Huang X Su X Fu Y Feng S Zhou Y Leukocyte-rich versus leukocyte-poor platelet-rich plasma and hyaluronic acid for knee osteoarthritis: a systematic review and network meta-analysis. J Orthop Surg Res. (2026) 21(1):222. 10.1186/s13018-026-06689-4 41629990 PMC13032500

[147] Rui S Dai L Zhang X He M Xu F Wu W Exosomal miRNA-26b-5p from PRP suppresses NETs by targeting MMP-8 to promote diabetic wound healing. J Control Release. (2024) 372:221–33. 10.1016/j.jconrel.2024.06.050 38909697

